# scDiffCom: a tool for differential analysis of cell–cell interactions provides a mouse atlas of aging changes in intercellular communication

**DOI:** 10.1038/s43587-023-00514-x

**Published:** 2023-11-02

**Authors:** Cyril Lagger, Eugen Ursu, Anaïs Equey, Roberto A. Avelar, Angela Oliveira Pisco, Robi Tacutu, João Pedro de Magalhães

**Affiliations:** 1 Integrative Genomics of Ageing Group, Institute of Life Course and Medical Sciences, University of Liverpool, Liverpool, UK; 2 Systems Biology of Aging Group, Institute of Biochemistry of the Romanian Academy, Bucharest, Romania; 3 Department of Medicine, Karolinska Institutet, Stockholm, Sweden; 4 Chan Zuckerberg Biohub, San Francisco, CA USA; 5 Present Address: Altos Labs, San Diego, CA USA; 6 Present Address: Insitro, Inc., South San Francisco, USA; 7 Present Address: Institute of Inflammation and Ageing, University of Birmingham, Birmingham, UK

**Keywords:** Computational biology and bioinformatics, Cell signalling, Ageing

## Abstract

Dysregulation of intercellular communication is a hallmark of aging. To better quantify and explore changes in intercellular communication, we present scDiffCom and scAgeCom. scDiffCom is an R package, relying on approximately 5,000 curated ligand–receptor interactions, that performs differential intercellular communication analysis between two conditions from single-cell transcriptomics data. Built upon scDiffCom, scAgeCom is an atlas of age-related cell–cell communication changes covering 23 mouse tissues from 58 single-cell RNA sequencing datasets from Tabula Muris Senis and the Calico murine aging cell atlas. It offers a comprehensive resource of tissue-specific and sex-specific aging dysregulations and highlights age-related intercellular communication changes widespread across the whole body, such as the upregulation of immune system processes and inflammation, the downregulation of developmental processes, angiogenesis and extracellular matrix organization and the deregulation of lipid metabolism. Our analysis emphasizes the relevance of the specific ligands, receptors and cell types regulating these processes. The atlas is available online (https://scagecom.org).

## Abstract (web summary)

Change in intercellular communication is an important but poorly characterized hallmark of aging. Here the authors provide a bioinformatics tool to infer changes in cell–cell signaling and an atlas of age-related communication changes in 23 mouse tissues.

## Main

Aging remains a poorly understood biological process despite affecting most organisms^1^. One of the difficult aspects to model is how the dynamics of molecular changes and tissue homeostasis influence each other throughout the lifespan. To gain further insights on how to bridge this gap, we focused our attention on intercellular communication (ICC). Dysregulation of ICC has been defined as a hallmark of aging^2,3^ and has recently been proposed as one of the causes leading to the cell-to-cell stochasticity arising with age^4^. Well-known communication deregulations include inflammaging (a chronic low-grade age-associated inflammation)^5^, impaired immune surveillance^6^, increase in senescence-associated secretory phenotype (SASP)^7^, altered communication between stem cells and their niche^8,9^, remodeling of the extracellular matrix^10,11^ and changes in endocrine and neuronal communication^12^. Interestingly, interventions involving extracellular signals have been shown to partially reverse some of the aging phenotypes. This includes targeting endocrine mediators such as insulin-like peptides and growth hormones^13,14^, the use of anti-inflammatory compounds^15–17^, heterochronic tissue transplants and heterochronic parabiosis^18–20^.

Direct measurement of ICC is complicated and usually depends on the type of mediators considered, such as surface receptors, soluble factors, extracellular vesicles^21^ or even mitochondria^22^. However, recent studies have shown that specific aspects of ICC can be inferred from single-cell gene expression data^23,24^. After the pioneer study that drafted the first comprehensive database of ligand–receptor interactions (LRIs)^25^, and based on the development of statistical tools dedicated to building cell-type to cell-type communication networks^26–34^, it is now becoming standard to perform ICC analyses alongside the workflow of single-cell transcriptomic studies.

In the context of aging, single-cell omics is a recent but expanding field^35,36^, with already a few investigations reporting changes in ICC. This includes articles on the mouse brain^37^, on the mouse mammary gland^29^, on several rat tissues^38^, on the primate cardiopulmonary system^39^ and on human skin fibroblasts^40^. However, the ICC analyses performed in some of those studies suffer from several limitations, as they rely on tools designed to detect interactions rather than to investigate how the interactions change between two biological conditions (for example, young/old and healthy/sick). Indeed, the main approach so far has been to detect interactions in young and old samples independently and then to focus on signals appearing or disappearing with age. As a result, this method does not account for interactions that are detected in both conditions but are, nevertheless, changing significantly; it also disregards the magnitude of the signal variation. More importantly, this approach lacks a statistical test to assess the significance of those changes and, thus, to evaluate if they occur due to noise or due to a true biological effect.

To alleviate such limitations, we built a statistical framework specifically designed to perform differential analysis in ICC. Our resulting R package, called scDiffCom, can be applied to any human or mouse single-cell RNA sequencing (scRNA-seq) dataset to analyze changes in ICC between two given conditions in a given tissue. scDiffCom includes a collection of approximately 5,000 curated LRIs that we retrieved from seven publicly available resources^26–32^. The typical output of the package is a table of detected cell-type to cell-type interactions indicating, in particular, their strength and how they are regulated between the two conditions of interest. To facilitate the interpretation of these results, we implemented an over-representation test to determine the dominant variations at the gene, cell-type or functional level. In addition, the package provides several visualization tools.

We used scDiffCom on several published scRNA-seq datasets from the Tabula Muris Senis (TMS) consortium^41^ and the Calico murine aging cell atlas^42^ to create scAgeCom, a large-scale atlas of age-related ICC changes across 23 mouse tissues. Samples obtained from male and female mice or from different experimental techniques were treated separately to avoid any confounding factors. By leveraging independent secretomics data from six cell lines, we confirmed that the interactions detected by scDiffCom using only gene expression are globally consistent with protein secretion profiles. The results are hosted and accessible via an online web application (https://scagecom.org) that contains both tissue-specific analyses and a global section summarizing changes shared across multiple tissues.

Our aging-related analysis supports previous knowledge regarding ICC, depicting a widespread upregulation of immune system processes and inflammation; a downregulation of extracellular matrix organization, growth, development and angiogenesis; and deregulation of lipid metabolism. We also report a generally complex sex-dependent regulation of ICC with age. Despite considerable differences across experimental techniques, we were able to predict some of the ligands, receptors and cell types that might play key roles in such dysregulation. Due to its generality and to the large number of tissues considered, we think that scAgeCom contains a large amount of valuable data waiting to be interpreted, which might provide the community with potential therapeutic targets and hypotheses regarding the relationship between aging and ICC.

## Results

### LRIs from existing databases

As with other methods analyzing ICC from scRNA-seq data, our approach first relied on the collection of LRIs. To maximize the variety of interaction types, we retrieved LRIs from seven publicly available resources: CellChat^26^, CellPhoneDB^27^, CellTalkDB^28^, NATMI/connectomeDB2020 (ref. ^29^), ICELLNET^30^, NicheNet^31^ and SingleCellSignalR^32^. From them, we built both human and mouse databases, keeping only curated interactions, converting human genes to mouse orthologs for human-only resources and including both simple and complex LRIs. Simple LRIs are interactions involving a single-gene ligand with a single-gene receptor—for example, *Apoe:Ldlr*. On the other hand, a complex interaction involves heteromeric ligands or receptors—for example, *Col3a1:Itga1-Itgb1*.

**Fig. 1:**
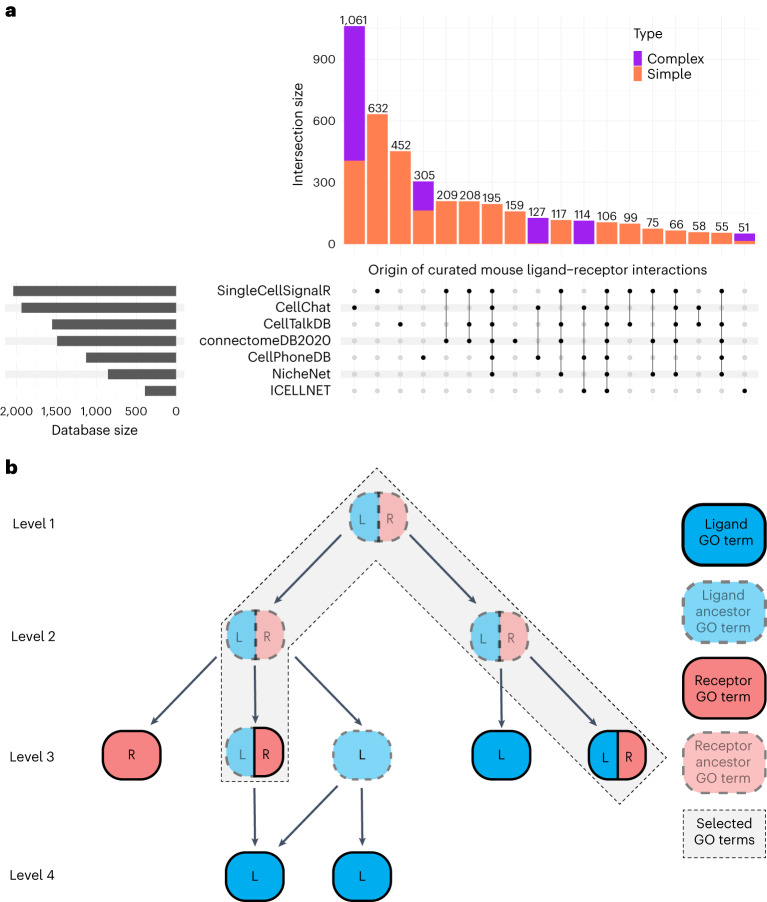
Origin and annotation of LRIs used by scDiffCom. **a**, Distribution of the 4,582 curated mouse LRIs classified according to the seven databases that they originated from. Each column of the UpSet plot corresponds to one intersection in an equivalent Venn diagram. Simple LRIs are composed of a single ligand gene and a single receptor gene, whereas complex LRIs contain a heteromeric ligand and/or a heteromeric receptor. Data are available in Supplementary Table 2. **b**, Method used to assign GO terms to a given LRI. Selected terms correspond to the intersection of the nodes of the two GO subgraphs made from the terms and ancestor terms of the ligand (blue) and receptor (red).

Our approach resulted in 4,582 mouse LRIs (simple: 3,479, complex: 1,103) and 4,785 human LRIs (simple: 3,648, complex: 1,137) directly accessible from our R package scDiffCom (Fig. 1a and Supplementary Tables 1 and 2). Whenever possible, we also included the sources used by each of the seven databases to curate their interactions, including references to PubMed identifiers, FANTOM5 (ref. ^25^), HPMR^43^, HPRD^44^, IUPHAR^45^, Reactome^46^ or KEGG^47^.

### Functional annotation of LRIs

We annotated all LRIs using a standardized and consistent framework. We first associated Gene Ontology (GO) terms^48^ to each interaction in a way that conveys biological meaning related as much as possible to the interaction itself rather than to each gene independently. Simply taking the intersection between the ligand GO terms and receptor GO terms would have resulted in a considerable number of empty intersections (as most genes are annotated with specific GO terms and not all parent terms). Instead, we associated to each LRI all GO terms formed by the intersection of two sets of nodes: (1) the nodes of the GO graph made of the ligand GO terms with their ancestors and (2) the nodes of the corresponding receptor GO graph (Fig. 1b). Because this method is prone to attaching lowly informative terms (namely, those near the root of the GO graph), we also computed and indicated the level of each GO term (namely, its depth in the GO graph) to facilitate downstream analysis. Then, we added KEGG pathways^47^ to each LRI if all genes present in the interaction were part of the pathway. All these annotations are directly accessible from scDiffCom (Supplementary Tables 3–6).

Anticipating our aging analysis, we also annotated the genes from the mouse LRI database with the number of PubMed articles associating each of these genes (or its human homolog) with aging or age-related diseases (excluding cancers). We also indicated if each gene (or its human homolog) is referenced in the GenAge^49^, CellAge^50,51^, LongevityMap^52^ or Gene Expression^53^ database of the Human Ageing Genomic Resources (HAGR)^49^. According to our search, the top 10 ligands/receptors associated the most with the aging literature are Apoe*, App*, *Snca*, Psen1*, *Ldlr*, Tnf*, Il6*, *Crp*, Tlr4* and *Il1b*, where a star indicates genes that are also referenced in the HAGR (Supplementary Table 7).

### Differential cell–cell communication analysis with scDiffCom

**Fig. 2:**
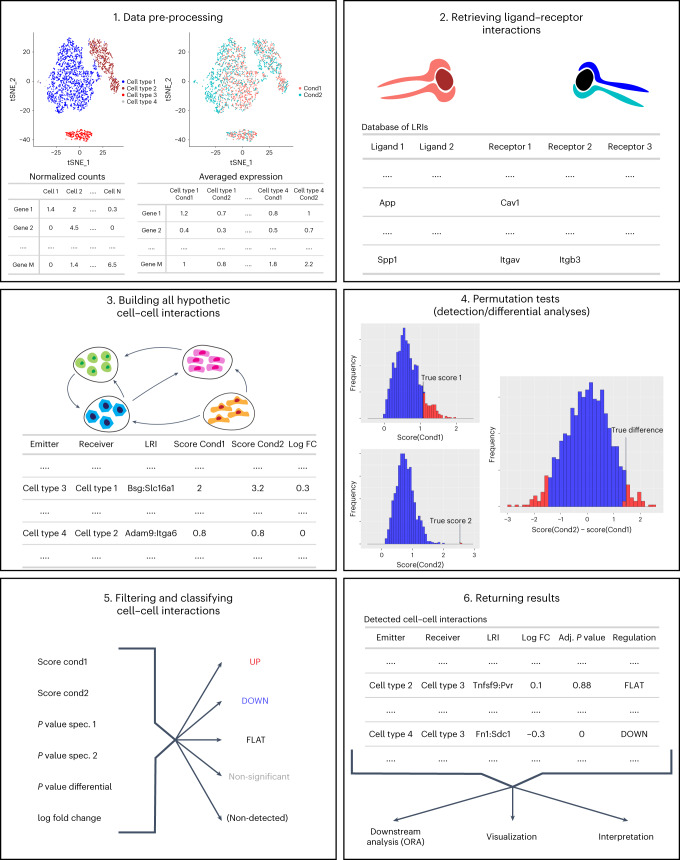
Workflow summary of scDiffCom. Read counts/UMIs from the single-cell dataset are aggregated by cell types and conditions (1). Genes are then joined with our database of LRIs (2) to build all the potential CCIs that can occur between cell types (3). Statistical permutation tests are then performed to evaluate the biological significance of each CCI and its differential expression (4). They are then classified based on several computed variables, such as their scores, *P* values and log fold change (5). Results are returned in a convenient format for downstream analyses and interpretation (6). FC, fold change; tSNE, t-distributed stochastic neighbor embedding.

We designed a bioinformatics method, available within the R package scDiffCom (https://github.com/CyrilLagger/scDiffCom), to detect cell-type to cell-type communication patterns that significantly change between two conditions in a given scRNA-seq dataset (Fig. 2). The dataset must be formatted as an R Seurat object^54–56^ and contain cells annotated by cell types and by the two conditions on which the differential analysis will be performed. The package then considers all possible interactions between each cell-type pair, based on the LRIs described in the previous subsection. We call each of those potential signals a cell–cell interaction (CCI). Each CCI is then assigned a score (independently in each condition) based on the average expression of the genes across the respective cells. As such, the CCI score should be interpreted as the typical interaction that would occur between two cells randomly picked from the pool of emitter, respectively receiver, cells. This score has the advantage of not depending on the number of cells captured, a quantity that is usually biased by current experimental protocols^57^. However, a limitation of this score is its inability to account for absolute communication across all cells and, consequently, to account for potential changes in cell composition.

Based on the CCI score, we first assess the biological relevance of each CCI (in each condition independently) by combining previously proposed approaches. To be considered detected, a CCI has to (1) not be lowly expressed; (2) be ‘specific’, as originally defined by the authors of CellPhoneDB^27,58^; and (3) have a large enough score (compared to the other remaining CCIs). The permutation test shuffles the cell-type annotation attached to each cell to estimate how specific to a given emitter–receiver cell-type pair a particular LRI is. This allows the removal of non-specific CCIs that are likely not biologically relevant.

Alongside the detection analysis, we implemented a second permutation test to assess if the score of each CCI significantly changes between the two conditions of interest. This consists of randomly exchanging the condition label of each cell to see whether the test statistic, namely the difference between the CCI scores of each condition, is different from zero. Choosing permutation tests was motivated by the fact that they are non-parametric—that is, they make no assumptions regarding the distributions of the underlying variables^59^. This was particularly useful in this context, as there is no obvious way to model the CCI score distribution. In addition, permutation tests can be applied to unbalanced and low sample size scenarios, which frequently appear in single-cell studies.

Finally, based on both the detection and differential analysis, each CCI is classified into one of four possible categories, further referred to as ‘regulation’. It can be upregulated (UP), downregulated (DOWN), stable (FLAT) or correspond to a non-significant change (NSC). As such, the main output of scDiffCom is a table that contains all detected CCIs with relevant information, including their regulation, log fold changes, adjusted *P* values and scores.

### Over-representation analysis to extract dominant changes

As it is typical to detect several thousands of CCIs in a single tissue, scDiffCom also performs an over-representation analysis (ORA) to extract the dominant differential patterns. Our method differs from usual enrichment analyses that typically infer GO terms associated with a list of genes of interest and, therefore, requires defining a background set of genes^60^. Here, the detected CCIs (as opposed to the genes) are classified into groups of interest (namely, UP, DOWN or FLAT) and a background (all other CCIs). Then, ORA measures the statistical association between the CCIs of interest and any feature that can be associated with them, such as GO terms and KEGG pathways, but also genes and cell types. As will be confirmed below, this approach also has the advantage of not being biased toward generic and lowly informative GO terms, despite the way that we annotated LRIs in the first place. Indeed, as being generic, these terms tend to be attached to most CCIs and, therefore, to have similar odds in both the group of interest and the background, making them rarely over-represented.

In practice, scDiffCom performs ORA on the following features by default: LRIs, individual ligands, individual receptors, emitter–receiver cell-type pairs, emitter cell types, receiver cell types, GO terms and KEGG pathways. Analyzing individual ligands is useful to detect those that may take part in various regulated CCIs involving different receptors (or vice versa). The same logic is valid for considering the cell types individually. Moreover, scDiffCom offers the opportunity to perform ORA on additional user-defined attributes. To facilitate the interpretation of these results, over-represented features can be sorted according to a score that combines the usual odds ratio (OR) and adjusted *P* value: ${ORA\; score}={-\log }_{2}\left({OR}\right)\bullet {\log }_{10}\left({adj}.{pval}\right)$. Finally, scDiffCom includes several built-in visualization functions that allow the user to easily take advantage of these statistical scores.

### scAgeCom: a mouse aging atlas of ICC

**Fig. 3:**
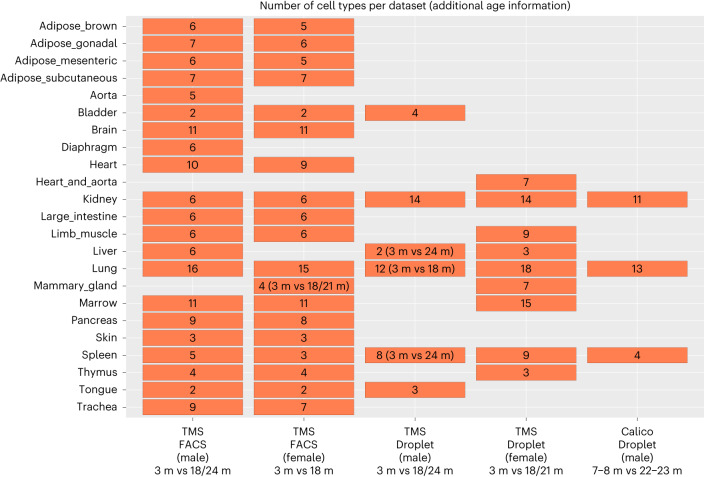
The 58 scRNA-seq aging datasets used to build scAgeCom. scAgeCom was built by applying scDiffCom to 58 public scRNA-seq aging datasets encompassing 23 tissues (*y* axis) from both male and female mice and different experimental techniques (*x* axis). The number of cell types detected per dataset is indicated in each rectangle. Information regarding the ages of the mice is indicated along the *x* axis and applies to all datasets of a given column unless specified otherwise in specific rectangles. Empty areas indicate unavailable data. Data are available in Supplementary Table 8. m, months.

We built a mouse atlas of age-related changes in ICC by applying scDiffCom (using 10,000 permutations and default parameters) to 58 scRNA-seq datasets from the TMS consortium^41^ and the Calico murine aging cell atlas^42^. The datasets cover 23 organs distributed across five categories (TMS FACS (male), TMS FACS (female), TMS Droplet (male), TMS Droplet (female) and Calico Droplet (male)) and contain both young and old samples (Fig. 3). When necessary, cell types were renamed or regrouped to facilitate the comparison across datasets. We also noticed that some important cell types were occasionally not captured in specific tissues (for example, adipocytes in adipose tissues) due to technical limitations. Notably, male and female samples were treated independently to avoid confounding effects that would otherwise be likely to arise, as we observed that cells were not uniformly distributed across sex, cell types and tissues (Supplementary Table 8). Keeping male and female samples separate also allowed us to perform a sex-differential analysis (Supplementary Text 1 and Supplementary Fig. 1) with the potential to reveal the effects of sex dimorphism on ICC aging.

**Fig. 4:**
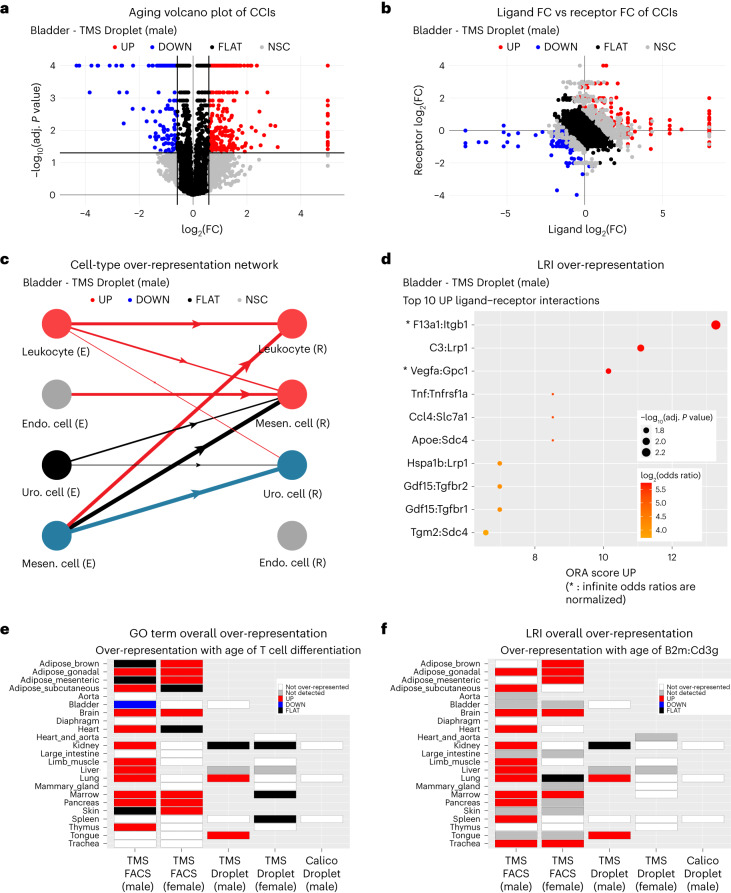
Typical figures available in scAgeCom. Various types of figures are available as part of our atlas scAgeCom and accessible online at https://scagecom.org/. A non-exhaustive list is provided here as an illustration. **a**, Volcano plots show how CCIs are distributed in terms of their aging log fold change, adjusted *P* value and regulation class (UP, DOWN, FLAT and NCS) for each specific dataset. Adjusted *P* values are computed by the scDiffCom two-sided permutation-based differential expression test. **b**, Ligand FC versus receptor FC plots indicate if the change with age of a given CCI score is driven by a change in the expression of the ligand, the receptor or both. **c**, Cell-type networks show which emitter cell types (E), receiver cell types (R) and cell-type pairs are over-represented as upregulated, downregulated, stable or both upregulated and downregulated with age. The latter scenario might occur when both upregulated and downregulated LRIs are significantly present between two cell types. **d**, ORA plots display the top over-represented terms of a given category (here, LRIs upregulated with age in the bladder) in terms of their OR, adjusted *P* value and ORA score computed from the two-sided Fisher’s exact test implemented in scDiffCom. **e**,**f**, Overall ORA plots summarize over-representation results of a given term from a category (here, **e**, the GO term ‘T cell differentiation’, and **f**, the LRI B2m:Cd3g) across all datasets. FC, fold change.

Our aging atlas can be accessed from an online application (https://scagecom.org/) that provides either results for each dataset independently or results summarizing the dominant age-related signals shared across tissues (Fig. 4). Over the 58 datasets, scDiffCom detected 393,035 CCIs regulated as follows with age: 5% UP, 13% DOWN, 56% FLAT and 26% NSC. The number of returned CCIs corresponds to an average of 100 detected LRIs (s.d. = 81) between any cell-type pair. Interestingly, 1,135 LRIs (out of 4,555) were never observed as part of any detected CCI (Supplementary Table 9), although their genes were individually detected in the scRNA-seq datasets. This illustrates the importance of the detection procedure to filter out interactions and minimize false discoveries.

**Fig. 5:**
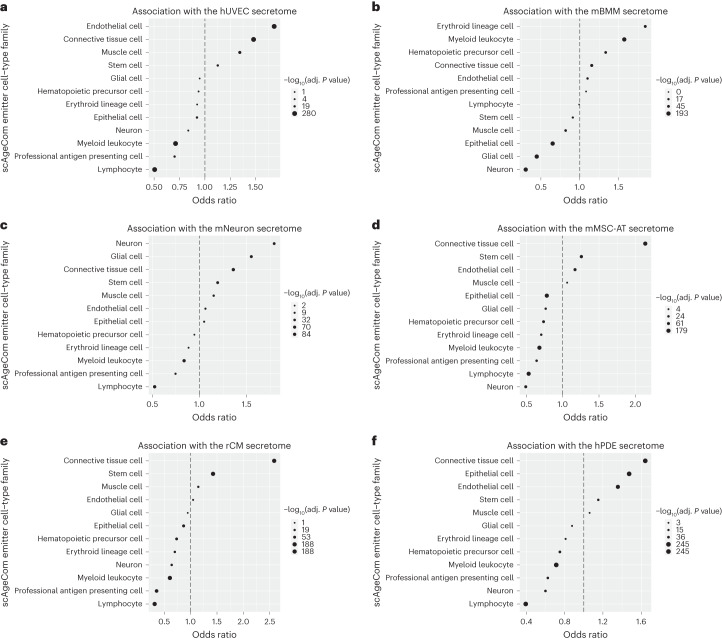
scDiffCom predicts CCIs globally consistent with secretomics data. CCIs detected by scDiffCom from transcriptomics data are globally consistent with the proteomics secretion profile of six specific cell lines. Each cell line has a strong and positive association with related cell-type families from scAgeCom as well as a strong and negative association with unrelated families. ORs and *P* values were computed from a two-sided Fisher’s exact test, and *P* values were further adjusted for multiple comparisons (Benjamini–Hochberg procedure). **a**, CCIs with a ligand that is part of the secretome from hUVECs are more likely emitted from endothelial or connective tissue cells, as opposed to immune cells. **b**, The secretome from mBMMs is positively associated with CCIs emitted from hematopoietic lineage. **c**, The secretome of mNeurons is mostly associated with CCIs emitted from brain cells. **d**, The secretome from mMSC-ATs is associated with CCIs emitted from connective tissue cells and stem cells. **e**, The secretome from rCMs is mostly associated with CCIs emitted from connective tissue cells and stem cells. **f**, The secretome from hPDE cells is mainly associated with CCIs emitted from connective tissue cells, epithelial cells and endothelial cells. Source data

**Extended Data Fig. 1:**
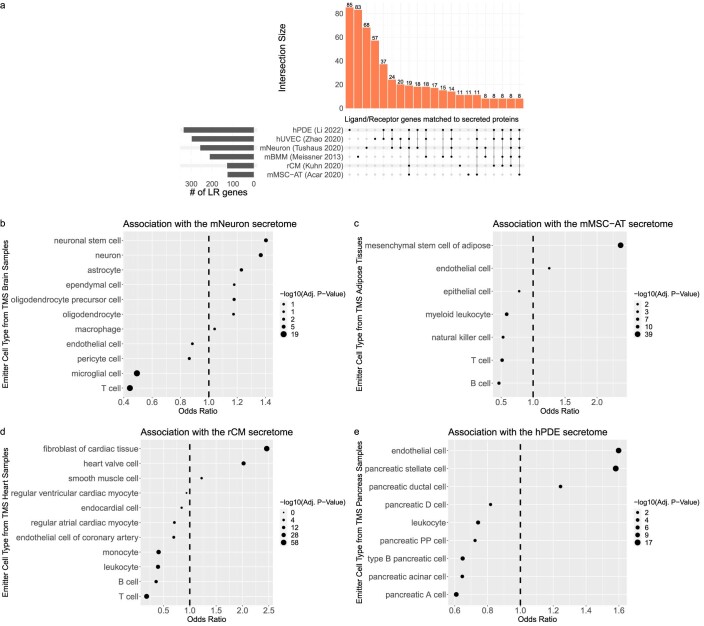
Consistency between secretomics data and tissue-specific scDiffCom detection results. **a**, After keeping the secreted proteins that matched to ligand and receptor genes present in the scDiffCom LRI database, mBMM, hPDE, mNeuron, and hUVEC cells have the highest number of specific proteins (that is only secreted by them), whereas rCM and mMSC-AT cells have most of their secreted proteins in common with other cell types; **b**-**e**, several secretomics datasets have their cell line originating from a tissue present in scAgeCom, allowing us to perform tissue-specific association analyses. Odds ratios and p-values were computed from a two-sided Fischer’s exact test and p-values were further adjusted for multiple comparisons (Benjamini-Hochberg procedure); **b**, for scAgeCom TMS Brain results, the secretome from mNeuron is predominantly associated with CCIs emitted from consistent cell types, namely neuronal stem cells and neurons; **c**, for scAgeCom TMS Adipose Tissue results, the secretome of mMSC-AT is predominantly associated with CCIs emitted from the corresponding cell type, namely mesenchymal stem cells; **d**, for scAgeCom TMS Heart results, the secretome of rCM is predominantly associated with CCIs emitted from heart-specific cell types (fibroblast of cardiac tissue and heart valve cell) but is neither positively nor negatively associated with regular ventricular cardiac myocytes; **e**, for scAgeCom TMS Pancreas results, the secretome of hPDE is predominantly associated with CCIs emitted from endothelial cells and pancreatic stellate cells and to a lesser extent with its corresponding cell type, namely pancreatic ductal cells. Source data

As scDiffCom is based on mRNA expression rather than protein expression, we employed multiple secretomics datasets to indirectly validate the biological significance and cell-type specificity of the CCIs identified in scAgeCom. We analyzed six proteomics datasets that characterize the secretion profiles of the following cell types cultured in vitro: mouse bone-marrow-derived macrophages (mBMMs)^61^, mouse neurons (mNeurons)^62^, mouse mesenchymal stem cells of adipose tissues (mMSC-ATs)^63^, rat cardiomyocytes (rCMs)^64^, human umbilical vein endothelial cells (hUVECs)^65^ and human pancreatic ductal epithelial (hPDE) cells^66^. We assessed the validity of scDiffCom by computing the association between these cell lines and the emitter cells in scAgeCom, based on if the ligands of the identified CCIs were secreted by the given cell line. Despite the inherent limitations of comparing proteomics to transcriptomics data, comparing data across different species and comparing cells from in vivo samples to cells cultured in vitro, we observed a significant level of consistency. For example, when considering CCIs whose ligand has been detected in the hUVEC secretome, the odds of their emitter cells being endothelial cells are 1.68 times greater (adjusted *P* < 1 × 10^−15^, Fisher’s exact test), in contrast to other cell types. Conversely, the odds of their emitter cells being lymphocytes are 1.98 times lower (adjusted *P* < 1 × 10^−15^, Fisher’s exact test) (Fig. 5a). A similar consistent association is confirmed for the other secretomics datasets and scAgeCom emitter cell-type families (Fig. 5b–f). This pattern can also generally be observed at the tissue-specific level when considering cell types rather than families (Extended Data Fig. 1). Overall, this gives further support that CCIs predicted by scDiffCom using only scRNA-seq likely correspond to actual protein-mediated extracellular signaling, even though transcriptome–proteome correlations might be modest.

**Extended Data Fig. 2:**
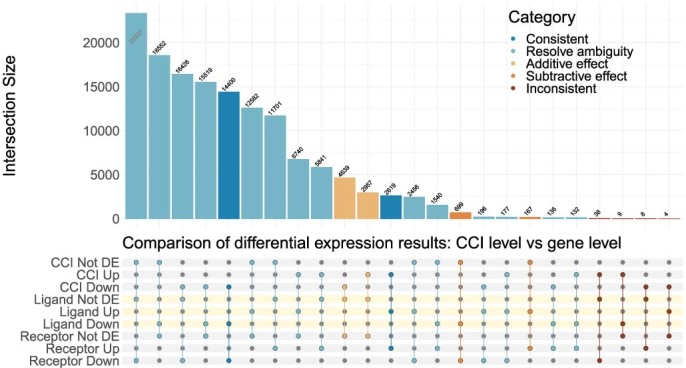
Comparing scDiffCom differential test to standard differential gene expression analysis. Using the 393,035 scAgeCom CCIs as a benchmark, the UpsetPlot shows how they are distributed in functions of scDiffCom differential analysis (rows 1-3), standard differential analysis of the ligand (rows 4-6), and standard differential analysis of the receptor (rows 7-9). For clarity, we removed the column counting CCIs not differentially expressed (Not DE) in all three cases. For most of the remaining CCIs, scDiffCom resolves the ambiguity that appears when performing differential analysis on the ligand and receptor individually. For example, scDiffCom considers that among the CCIs with a non-differentially expressed ligand but a down-regulated receptor, 23,337 of those are not differentially expressed whereas 16,426 are down-regulated. We also observed additive effects (CCI differentially expressed while both the ligand and receptor are not) or subtractive effects (CCI not DE while both the ligand and receptor are DE in the same direction). Additive effects can be easily explained by the CCI having a fold change larger than the threshold while this is not the case for its genes individually. Subtractive effects are rare and mainly due to the p-value of the CCI being not significant, despite ligand and receptor p-values being significant. Inconsistent cases only represent a marginal fraction of all cases and can be explained by CCIs and genes having fold-changes and p-values close to the thresholds. Source data

In addition to its detection capabilities, we also assessed several other aspects of scDiffCom using scAgeCom results as a benchmark. First, we confirmed the relevance of performing the differential analysis test on the CCI score that combines the expression of the ligand and receptor rather than performing a standard differential test on each gene separately. By comparing these two approaches on all scAgeCom CCIs, we observed that the regulation of a considerable fraction of them was determined without ambiguity by scDiffCom but not by standard differential analysis (Extended Data Fig. 2). Second, we confirmed that scDiffCom does not introduce a bias toward generic GO terms when performing ORA (Supplementary Text 2 and Supplementary Figs. 2 and 3). Finally, we confirmed the importance of the prior knowledge contained in LRI databases to characterize CCIs from scRNA-seq data (Supplementary Text 3 and Supplementary Fig. 4).

### Aging dysregulates several aspects of cell–cell communication

**Fig. 6:**
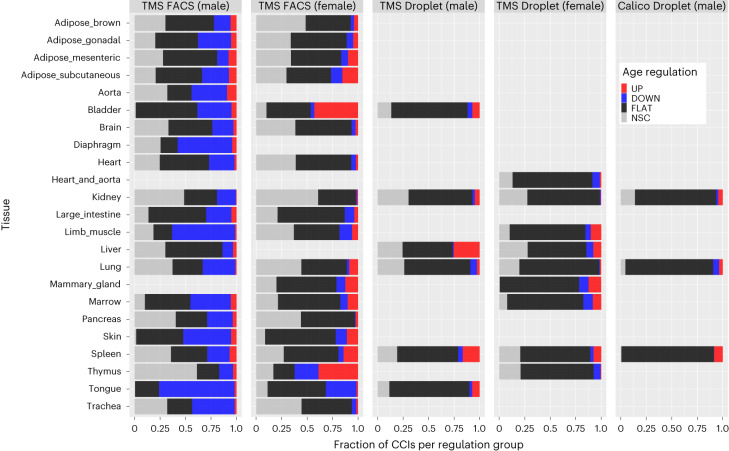
Distribution of the scAgeCom CCIs in terms of age regulation per dataset. The percentage of CCIs being upregulated (UP), downregulated (DOWN), stable (FLAT) or changing non-significantly (NSC) with age varies across datasets in functions of the tissue, sex and experimental techniques. We observed that TMS FACS datasets are generally more noisy (that is, have a higher percentage of NSC CCIs) than Droplet datasets. TMS FACS (male) datasets also admit a higher percentage of downregulated CCIs with age compared to all other conditions, in agreement with the general decrease in gene expression previously reported in these samples^41^. Source data

The regulation of the CCIs with age strongly varies across datasets, sex and experimental techniques (Fig. 6). The tissues from TMS FACS (male) clearly show a larger fraction of downregulated CCIs compared to all other datasets, including those from TMS FACS (female). We also observed more variability and noise (namely, more NSC CCIs) in FACS compared to Droplet datasets. These general observations are partially explained in the discussion below. Then, as it was not possible to present all results contained in scAgeCom, we focused our attention on age-related changes that we considered of primary interest because they were shared across several tissues, involving genes not previously associated with aging, involving genes detected in secretomics datasets or showing interesting sex-dependent patterns.

**Extended Data Fig. 3:**
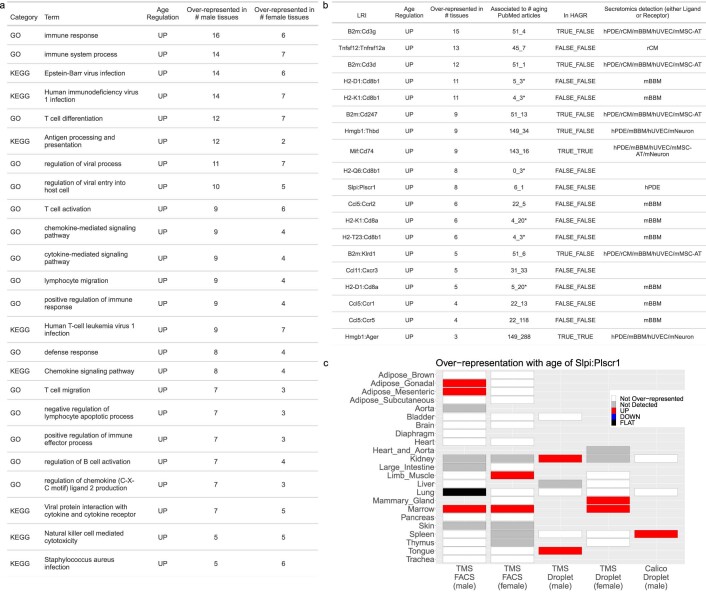
Processes and LRIs related to inflammation, the immune system, and viral processes are globally over-represented as up-regulated with age across tissues. **a**, List of selected GO terms and KEGG pathways over-represented as up-regulated with age in several male and female tissues. **b**, List of selected LRIs over-represented as up-regulated with age in several tissues. We indicate how many aging PubMed articles mention individual genes and if each gene is part of the Human Ageing Genomic Resources (HAGR). Of note, the fact that histocompatibility 2 (H2) genes were only associated with a low number of aging PubMed articles (as indicated with a star) is technical rather than biological and because most of these genes are not often discussed individually but rather as a family in the literature. The column Secretomics detection indicates if at least one of the genes of a given LRI has been detected in some of the six secretomics datasets we analyzed. **c**, Over-representation results for the LRI Slpi:Plscr1 across the 58 scAgeCom datasets. It is over-represented as up-regulated with age in 10 datasets and consistently in the three bone marrow datasets.

Consistent with the literature, we first observed a major upregulation of inflammatory, immune system and viral processes, as revealed by the numerous related GO terms, KEGG pathways and immune cell types over-represented across several tissues (Extended Data Fig. 3a). From the gene perspective, the interactions that are over-represented in the highest number of tissues include *B2m:Cd3g*, *B2m:Cd3d*, *Tnfsf12:Tnfrsf12a*, *H2-D1/K1/Q6:Cd8b1*, *Mif:Cd74*, *Hmgb1:Thbd*, *Slpi:Plscr1* and *Ccl5* interacting with different chemokine receptors, such as *Ccrl2*, *Ccr1* and *Ccr5* (Extended Data Fig. 3b). As a protein, B2M was observed in the secretome of five of the six cell lines that we analyzed, further supporting a tissue and cell-type-independent role. Leveraging our annotation of LRIs with PubMed articles on aging, we also noticed that *Slpi:Plscr1*, although being over-represented as upregulated in eight tissues, is an interaction whose genes have almost not been studied in the context of aging. We, therefore, provide further interpretation regarding its potential role in aging in our discussion below.

**Extended Data Fig. 4:**
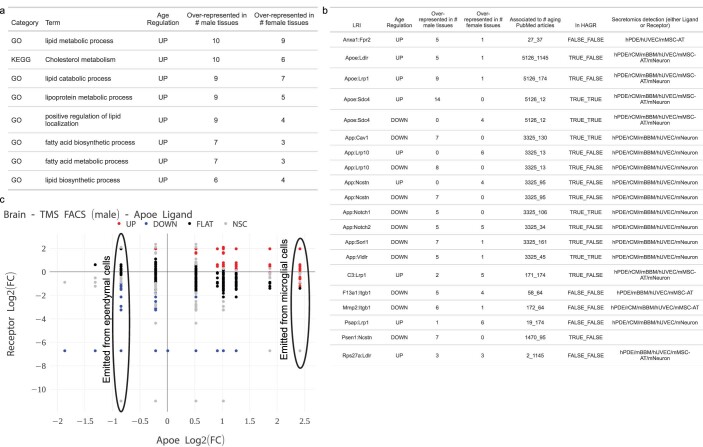
Processes and LRIs related to lipid metabolism are over-represented as globally dysregulated with age across tissues. **a**, List of selected GO terms and KEGG pathways over-represented as up-regulated with age in several male and female tissues. **b**, List of selected LRIs over-represented as either up or down-regulated with age in several tissues. The column Secretomics detection indicates if at least one of the genes of a given LRI has been detected in some of the six secretomics datasets we analyzed. Several LRIs display opposite aging patterns in male compared to female datasets. **c**, Comparison between the fold changes of the ligand and the receptor for the CCIs from the TMS FACS (male) Brain dataset that have Apoe as ligand. Changes in Apoe expression with age (x-axis) are strongly cell-type-specific. For example, Apoe is most strongly up-regulated in microglial cells while it is down-regulated in ependymal cells.

Lipid metabolism is another process dysregulated in many tissues with age. Several related GO terms and KEGG pathways are globally over-represented as upregulated (Extended Data Fig. 4a). However, looking at over-represented LRIs revealed more complex patterns, such as the upregulation of CCIs involving *Apoe* in male tissues and *App* in female tissues but the downregulation of CCIs involving *Apoe* in female tissues and *App* in male tissues (Extended Data Fig. 4b). Understanding such patterns in detail requires going beyond the over-representation results and exploring the specific CCIs of each tissue. For example, focusing on male brain samples, we observed that changes in CCIs involving *Apoe* are cell-type dependent (Extended Data Fig. 4c), typically upregulated when emitted from microglial cells but downregulated when emitted from ependymal cells. Both *App* and *Apoe* are associated with a large number of PubMed articles on aging, essentially for their relevance in Alzheimer’s disease. However, our results suggest that these two proteins might play important roles in aging and ICC outside of the central nervous system as well, as they appear in dysregulated CCIs across many tissues and as they are detected in the secretome of most of the cell lines that we analyzed (Extended Data Fig. 4b).

**Extended Data Fig. 5:**
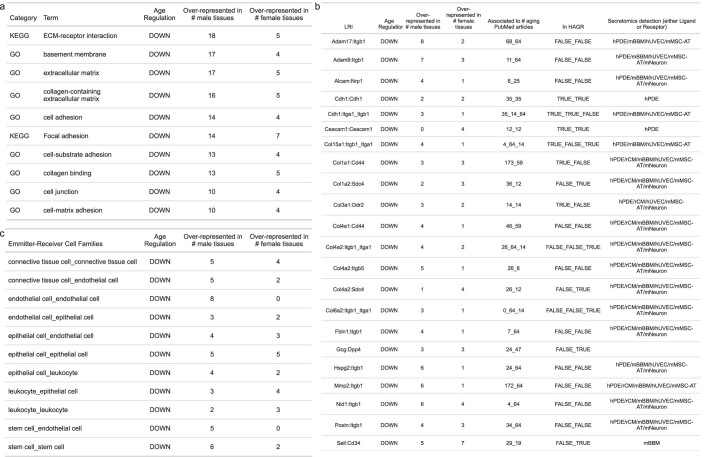
Processes, LRIs, and cell-type families related to the extracellular matrix and adhesion processes are over-represented as globally down-regulated with age across tissues. **a**, List of selected GO terms and KEGG pathways over-represented as down-regulated with age in several male and female tissues. **b**, List of selected LRIs over-represented as down-regulated with age in several tissues. The column Secretomics detection indicates if at least one of the genes of a given LRI has been detected in some of the six secretomics datasets we analyzed. **c**, Emitter-Receiver cell-type family pairs that are mainly over-represented as down-regulated with age in several tissues.

**Extended Data Fig. 6:**
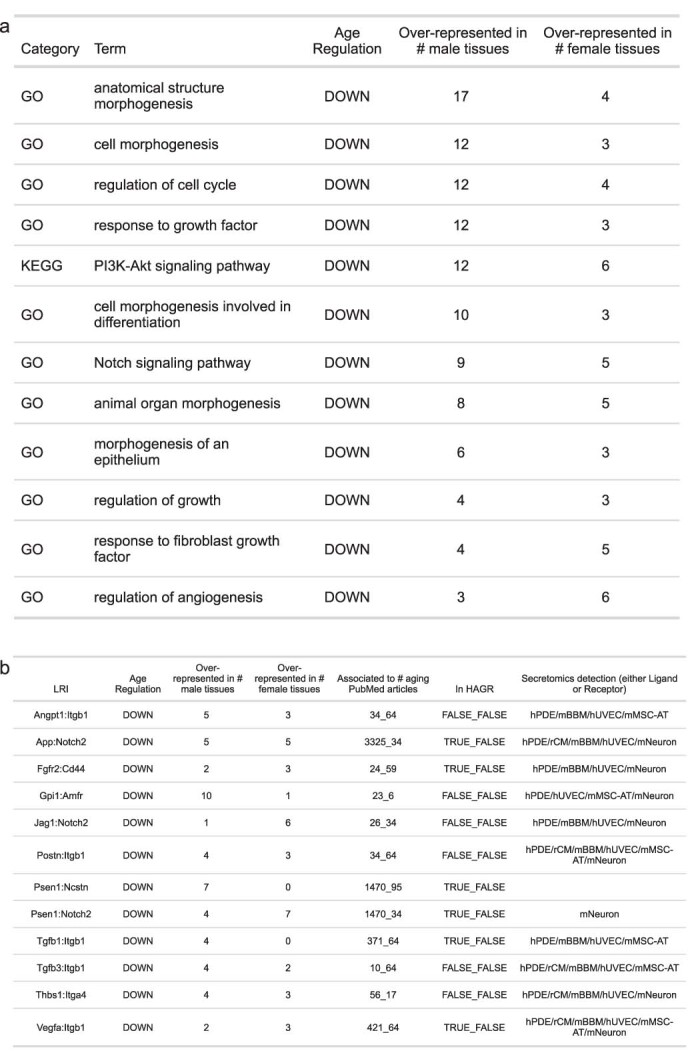
Processes and LRIs related to morphogenesis, development, and angiogenesis are over-represented as globally down-regulated with age across tissues. **a**, List of selected GO terms and KEGG pathways over-represented as down-regulated with age in several male and female tissues. **b**, List of selected LRIs over-represented as down-regulated with age in several tissues. The column Secretomics detection indicates if at least one of the genes of a given LRI has been detected in some of the six secretomics datasets we analyzed.

The most over-represented processes among signals downregulated with age are related to the extracellular matrix and adhesion (Extended Data Fig. 5a). The main corresponding LRIs involve collagens, cadherins and metallopeptidases interacting mainly with integrins (Extended Data Fig. 5b), mostly among connective tissue cells, epithelial cells and endothelial cells (Extended Data Fig. 5c). In addition to the changes undergone by the extracellular matrix, interactions related to growth, development, survival, differentiation and angiogenesis are also significantly downregulated with age (Extended Data Fig. 6). Cell-type pair over-representation also indicates the downregulation of communication among stem cells and from stem cells toward endothelial cells (Extended Data Fig. 5c).

In addition to these widespread age-related changes, scAgeCom contains much more specific information that cannot be detailed here but is available to the community from our atlas. Several large-scale downstream analyses are further possible to perform, such as combining scAgeCom with the results from the sex-differential analysis (Supplementary Text 1) to reveal patterns of sex dimorphism in ICC aging. As an illustration, we observed from the TMS FACS Lung data that 13% of the differentially expressed CCIs had a complex pattern, namely being more expressed in young males than in young females, decreasing with age in males and increasing with age in females. Involved in these CCIs are bronchial smooth muscle cells (64%), *App* (21%), *Pecam1* (4%), *Tgm2* (4%), *Itgb1* (22%), *Lrp10* (4%), *Mcam* (4%) and *Nrp1* (4%). Sex differences in airway remodeling and inflammation have been recently reviewed, highlighting sex-related and age-related lung modifications, such as surfactant secretion^67^. We expect that other similar complex patterns could be mined from scAgeCom in the future.

## Discussion

We provide a package to perform differential ICC analysis as well as a comprehensive database of age-related mouse CCIs. From a technical point of view, our results illustrate the importance of performing a proper statistical analysis when comparing intercellular signals extracted from scRNA-seq data. Using scAgeCom as a benchmark, variability and noise were indeed responsible for the classification of 26% of the detected CCIs as NSC interactions, namely those with a fold change larger than 1.5 but a non-significant adjusted *P* value. Had we not used a statistical test and based our analysis solely on the appearance and disappearance of CCIs between the two conditions, as in some previous studies, 82.4% of those NSC signals (that is, 84,998 CCIs) would have been falsely considered to be age regulated. Moreover, such an approach would have missed all CCIs detected in both conditions but showing a significant change with age—18,146 interactions, in our case.

We observed considerable disparities in the results depending on experimental techniques and sex (Fig. 6). Previous comparisons of single-cell sequencing techniques^68^ have claimed that droplet-based methods using unique molecular identifiers (UMIs) are less subject to amplification noise than Smart-seq2, potentially explaining why we observed fewer NSC CCIs in TMS Droplet and Calico Droplet than in TMS FACS. The other differences can be explained by several factors. First, different datasets sometimes compare different age groups (Fig. 3). Second, due to experimental limitations, captured cell types are rarely the same between two datasets of the same tissue. For example, for the Lung, there are 16 cell types in TMS FACS (male) against 12 cell types in TMS Droplet (male), resulting in more detected CCIs in the former than in the latter dataset (23,200 versus 11,711) and with a different distribution of the percentage of UP/DOWN/FLAT/NCS CCIs (Fig. 6). We also emphasize that the pronounced downregulation observed in TMS FACS (male) datasets (Fig. 6) was reported in a previous study performed on TMS datasets^69^.

We now provide more specific interpretations of the main biological results reported above. Regarding the increase of immune response processes with age, we observed a widespread upregulation of CCIs involving the ligand *B2m* (β2-microglobulin). *B2m* has already been recognized as a gene consistently overexpressed with age^53^ and as a pro-aging circulating factor whose elevated level negatively affects cognitive functions and neurogenesis in the mouse hippocampus^70^. Our results indicate that the increased secretion of the ligand *B2m* with age is systemic and appears to target T cells via their receptors *Cd3g*, *Cd3d* and, to a lesser extent, *Cd247* (explaining the occurrence of the GO term ‘T cell differentiation’). This could be a sign of increased antigen presentation in major histocompatibility complex (MHC) class I. Such a widespread pattern might also indicate detrimental effects of *B2m* not limited to the brain and reinforce the idea that this protein might be a potential therapeutic target, as previously suggested^71^.

Regarding the increase in inflammation with age, our results show a global over-representation of the terms ‘cytokine-mediated pathway’ and ‘chemokine-mediated pathway’ and of the ligands *Ccl5*, *Mif* and *Hmgb1*. Those ligands are known SASP factors, but we cannot say from our analysis whether they originate in senescent cells. The original analysis performed on the Calico data found very few cells expressing markers of senescence and no significant changes in aged tissues^42^. The original TMS study reported a higher fraction of cells expressing senescence markers in some (but not all) older age groups^41^, although the number of such cells was also very small (unpublished data). Follow-up studies, such as a cross-analysis between scAgeCom and the SASP atlas^72^, might allow us to explore such senescence-related hypotheses more precisely. Here, we only mention that the two most over-represented upregulated LRIs that we reported earlier, *Mif:Cd74* and *Hmgb1:Thbd*, actually seem to play compensatory roles in the context of senescence and inflammation. Indeed, it has been reported that macrophage migration inhibitory factor (Mif), a pleiotropic cytokine, can prevent cellular senescence and rejuvenate mesenchymal stem cells from age-induced senescence via CD74/AMPK/FOXO3a and autophagy in both rats and humans^73,74^. Along the same line, the high-mobility group box chromosomal protein 1 (Hmgb1) has pro-inflammatory effects when binding to RAGE/Ager^75^, but its sequestration by thrombomodulin acts as an anti-inflammatory mechanism^76^. According to our results, *Hmgb1:Thbd* is over-represented as upregulated in nine tissues and *Hmgb1:Ager* in three tissues (Extended Data Fig. 3b), pointing toward a global over-emission of *Hmgb1* with age that tissues might try to compensate by over-expressing *Thbd*.

Our analysis also revealed widespread upregulation of the LRI *Slpi:Plscr1* whose genes have received little attention in the context of aging. Secretory leukocyte peptidase inhibitor (SLPI) has anti-inflammatory, anti-bacterial, anti-fungal and anti-viral activities^77^, and its interaction with phospholipid scramblase 1 (PLSCR1) was suggested to inhibit HIV-1 infection of T cells^78^. In scAgeCom, *Slpi:Plscr1* is notably upregulated with age in all three bone marrow datasets (Extended Data Fig. 3c). Therefore, the increase with age in such communication patterns might represent a response to the latent and persistent invasion of the immune system by pathogens such as cytomegaloviruses, which can use the bone marrow as a reservoir of latency^79^.

Changes in lipid metabolism with age are known to have an important impact on the lifespan and age-related diseases^80^. Our most intriguing results concern the widespread sex-dependent deregulation of *Apoe*, its receptors and *App*. We indeed observed a general over-emission of *Apoe* and under-emission of *App* in most TMS FACS (male) tissues but an opposite trend in TMS FACS (female) tissues (Extended Data Fig. 4b). In the brain, these genes are known to play a role in Alzheimer’s disease, for which there are well-documented and important sex differences^81^. *App* is the precursor of amyloid beta (Aβ) peptides^82^; *Apoe* is a known regulator of Aβ clearance^83^; and interactions between *App* and *Apoe* receptors influence Aβ metabolism and toxicity^84,85^. Much less research has been performed on the function of *App* outside the central nervous system^86^, but some studies point toward its role in other pathologies, such as in obesity^87,88^, and in different tissues, such as skin^89^, intestine^90^ (where it is modulated by diet^91^) and muscles^92^, particularly at neuromuscular junctions^93^. Taken together, those studies and our results suggest that changes with age in *App* intercellular trafficking might lead to a variety of tissue-specific diseases.

The two main downregulated processes reported in our results—namely, changes in the extracellular matrix and development/growth/proliferation—are partially interdependent. They have in common the under-expression of integrins, which typically act as bidirectional mediators between the cytoplasm and the extracellular space and regulate mechanisms such as cell migration, adhesion, proliferation, apoptosis, tumor progression and senescence^94^. The multiple functions of these proteins and their deregulation that we observed across multiple organs (most notably when they interact with collagen) suggest that they might play key roles in the structural decline of tissues with age. They are also consistently downregulated in signaling involving stem cells, potentially impacting their maintenance and homeostasis, as suggested in previous articles^10^. Changes in processes related to growth and development also fit the hypotheses that the run-on from developmental mechanisms and programs may later impact on aging^95^.

A subpart of the development/growth/proliferation pathway concerns the global downregulation of angiogenesis and qualitative changes occurring in vessels. Several studies previously reported a decline with age in capillary density and in the formation of new blood vessels^96,97^, even leading some authors to postulate an ‘angiogenesis hypothesis of aging’^98^. Our analysis reveals that the pair *Gpi1:Amfr*, mainly detected from or to endothelial cells, is over-represented among downregulated CCIs in 10 male tissues (Extended Data Fig. 6b). Glucose-6-phosphate isomerase functions as an autocrine motility factor that stimulates endothelial cell motility^99^. This LRI could be an important regulator of microvascular aging, and its therapeutic potential seems worth investigating. Moreover, we found downregulation of *Angpt1* (angiopoietin-1) in several tissues (Extended Data Fig. 6b), which could lead to the formation of leaky vessels, as vessel stability relies on the balance of Angpt1 and Angpt2 (ref. ^100^).

We conclude with some of the limitations of using gene expression of ligands and receptors to infer ICC activity. First, using mRNA counts as a proxy for the actual level of secreted proteins may lead to an overestimation of some intercellular interactions, as we assume that all transcripts participate in signaling, even though a fraction of them might be produced for intracellular processes. Despite this concern, scDiffCom was still able to predict CCIs that were globally consistent with secretomics data. Second, it is not always clear how some interactions (for example, *App:Lrp10*) are shared between cells and which fraction of them are autocrine rather than paracrine. Third, by looking only at ligands and receptors, our method does not assess changes in other players acting in downstream signaling. Fourth, we did not consider all possible types of intercellular mediators, and we did not investigate inter-tissue interactions, such as endocrine signals. Fifth, the CCI score does not account for potential changes in cell composition. Despite these limitations, we think that our atlas will be useful for the community and lead to hypotheses on ICC and aging to be further validated.

## Methods

### Retrieving LRIs

We downloaded LRIs from seven publicly available databases. Data from CellChat (version 1.1.3), NicheNet (version 1.1.1) and SingleCellSignalR (version 1.10.0) were directly accessed from their associated R packages. Data from CellPhoneDB, CellTalkDB, connectomeDB2020 and ICELLNET were retrieved online (as of 16 April 2022) from their respective websites. All details regarding download dates and links are directly accessible from our package scDiffCom. We initially also retrieved LRIs from an eighth database, LRBaseDb from scTensor^33^, but we did not consider it further, as all curated LRIs present in it were derived from the seven others mentioned above.

### Processing LRIs

We analyzed the documentation and annotations of each resource to keep only their curated LRIs. We removed the interactions that were only bioinformatically predicted, such as from protein–protein interaction networks. We checked that gene symbols were approved by the HUGO Gene Nomenclature Committee (HGNC)^101^ and, when mouse data were available, approved by Mouse Genome Informatics (MGI)^102^. If mouse LRIs were not provided (namely, for CellPhoneDB, connectomeDB2020, ICELLNET, NicheNet and SingleCellSignalR), we converted human LRIs to their mouse equivalent by retrieving orthology information from Ensembl version 102 (ref. ^103^), accessed through the R package biomaRt (version 2.50.2)^104^. We only kept mouse LRIs whose genes had high-confidence homolog pairs (setting the parameter mmusculus_homolog_orthology_confidence to 1) or whose genes were already included in the two databases providing mouse LRIs directly (CellChat and CellTalkDB).

LRIs from each resource were combined in a single list. Special care was taken to avoid duplicates arising from the same interactions available for both directions (for example, G1:G2 versus G2:G1), typically for juxtacrine signaling where the notion of ligand or receptor can sometimes be arbitrary.

As some of the resources provide only simple interactions, some of their LRIs could be incomplete. To partially correct this effect, we removed simple LRIs present in such databases if they were also found in complex databases but only in a complex form. For instance, we removed *Col3a1:Itgb1*, existing in SingleCellSignalR, as it always appears in a complex form in CellPhoneDB, such as in *Col3a1:Itga1-Itgb1*.

We manually verified the combined list of LRIs and removed approximately 200 records that we considered mis-curated—for example, *Mapk1:Fgfr2*, *Calm1:Adcy8*, *Gnas:Adcy1* and *Hsp90aa1:Cftr*. To do so, we first annotated each gene with descriptions from MyGene.Info (version 3.2.2)^105,106^ and categories from OmniPath (version 3.3.13)^107^. We then identified genes that seemed unlikely to participate in ICC because they were not annotated as being potentially secreted and observed extracellularly. Finally, we explored the interactions involving those genes and evaluated the evidence supporting their existence. Some of the removed LRIs were initially included based on publications describing intracellular interactions that had been misinterpreted as intercellular—for example, Loo et al.^108^ is presented as evidence for *Hsp90aa1*:*Cftr*, but the article does not mention ICC.

### Annotating LRIs with GO terms and KEGG pathways

LRIs were annotated with GO terms by following a graph-based approach (Fig. 1b). GO terms associated with each gene were retrieved from Ensembl version 102 (ref. ^103^), accessed through the R package biomaRt (version 2.50.2)^104^. For a given LRI, we used the R package ontoProc (version 1.16.0)^109^ to build the ligand and receptor ontology subgraphs, whose nodes included their respective GO terms and ancestors up to the root node. For complex LRIs including, for example, multiple ligand genes, we considered the union of the terms associated with each ligand gene. The final LRI GO terms were those present in both the ligand and the receptor subgraphs—that is, the intersection of those graphs at the node level. scDiffCom can provide other GO term annotation methods (Supplementary Text 2), but we do not recommend them outside benchmarking purposes as we consider them less biologically relevant.

To annotate LRIs with KEGG pathways, we used the R package KEGGREST (version 1.34.0)^110^ to retrieve all pathways associated with a given gene. For each LRI, we then only retained the pathways that included both the ligand gene(s) and receptor gene(s).

### Annotating ligands and receptors with aging resources

To annotate the genes from the mouse LRI database with the number of PubMed articles associating them with aging, we first downloaded the gene2pubmed table from ftp://ftp.ncbi.nlm.nih.gov/gene/DATA/gene2pubmed.gz on 16 April 2022. We filtered the table by keeping only mouse and human genes and by removing articles associated with more than 50 genes. Second, we performed a PubMed search with the R package rentrez (version 1.2.3)^111^ for all articles containing at least one of the following keywords in their title or abstract (TIAB search): aging, longevity, senescence, age-related, dementia, alzheimer, parkinson, atherosclerosis, stroke, arthritis, osteoporosis and cataract. Diseases without a very direct and strong connection with aging, as well as cancers that have a too-broad scope, were excluded. Finally, we merged the gene2pubmed table with our aging search to count how many PubMed articles mentioning a given mouse ligand or receptor (or its human homolog) were related to aging.

To further determine if a gene of the mouse LRI database (or its human homolog) was previously associated with aging, we downloaded GenAge (human and mouse genes, build 20), LongevityMap (build 3) and the Microarray meta-analysis of Ageing Gene Expression database from the HAGR website (https://genomics.senescence.info/download.html) on 16 April 2022. On the same date, we retrieved the CellAge database of cellular senescence genes (build 2) from the supplementary material of Tejada-Martinez et al.^51^.

### CCI score based on the geometric mean

For each CCI in the form (emitter cell type, receiver cell type, ligand(s) and receptor(s)), a score $\varphi$ is computed in each condition as the geometric mean $\varphi =\sqrt{{e}_{L}\cdot {e}_{R}}$ between the averaged expression ${e}_{L}$ of the ligand gene in the emitter cells and the averaged expression ${e}_{R}$ of the receptor gene in the receiver cells (based on normalized non-log-transformed read counts/UMIs). In the case of complex LRIs with multiple ligand genes (or receptor genes) involved, ${e}_{L}$ (or ${e}_{R}$) is given by the minimum value from the set of average expressions of those genes.

Defining a CCI score is a standard approach when investigating ICC from scRNA-seq data^23,24^, although the way of computing the score varies between studies. Here, the choice of the geometric mean (similar to SingleCellSignalR) rather than the arithmetic mean (as used by CellPhoneDB) is motivated by several advantages. The first one is that the geometric mean tends toward zero if either ${e}_{L}$ or ${e}_{R}$ tends to zero. This implies that when a highly expressed ligand is combined with a lowly expressed receptor (or vice versa), the score is not dominated by the large ligand value, as would have been the case with the arithmetic mean. Along the same line, although transcript counts or UMIs give only an indirect representation of protein levels^112^, molecular interactions are usually modeled by the law of mass action^113^, which is by essence multiplicative and not additive in protein concentrations. Finally, the geometric mean provides a clear interpretation of the log fold change of the scores between the two conditions of interest. Indeed, we see that the log fold change of the CCI score across two hypothetical conditions $A$ and $B$ corresponds to the (arithmetic) average between the respective ligand and receptor log fold changes, $\log \left(\frac{{\varphi }_{B}}{{\varphi }_{A}}\right)=\log \left(\frac{\sqrt{{e}_{L,B}{e}_{R,B}}}{\sqrt{{e}_{L,A}{e}_{R,A}}}\right)=\frac{1}{2}\left(\log (\frac{{e}_{L,B}}{{e}_{L,A}})+\log (\frac{{e}_{R,B}}{{e}_{R,A}})\right)$.

At present, scDiffCom calculates CCI scores exclusively from gene expression and relies only on the LRI database as a prior source of information. However, additional forms of prior knowledge, such as binding kinetics parameters, may become available soon and enable more precise score calculations. Supplementary Text 4 provides some concrete examples.

### CCI detection and differential analyses

Our approach relies on three permutation tests to assess if a CCI is (1) cell-type pair specific in condition $A$, (2) cell-type pair specific in condition $B$ and (3) differentially expressed between $A$ and $B$. To be computationally more efficient, the three tests are done together as part of a single iteration loop. All threshold parameters described below can be adjusted by the users.

Given $m$ cell types and $l$ LRIs that are found in the scRNA-seq dataset, scDiffCom builds a table of ${m}^{2}\cdot l$ hypothetical CCIs. For each CCI, we compute the CCI scores ${\varphi }_{A}$ and ${\varphi }_{B}$, the log fold change $\log ({\varphi }_{B}/{\varphi }_{A})$ and the variables ${n}_{i,\,j}$ and ${d}_{i,\,j}$ corresponding to the number and fraction of emitter cells expressing the ligand ($i=L$) or receiver cells expressing the receptor ($i=R$) in either condition $(\,j\in \{A,B\})$. A CCI is deemed ‘not expressed’ in condition $j$ if $\left(\right.{n}_{L,\,j} < 5$ or ${n}_{R,\,j} < 5\left)\right.$ and $\left({d}_{L,\,j} < 0.1\right.$ or $\left.{d}_{R,\,j} < 0.1\right)$.

Only the CCIs ‘expressed’ in at least $A$ or $B$ are passed to the iteration loop. At each iteration $k$, three independent operations are done: (1) shuffling the cell-type labels of cells from condition $A$ and returning the random score ${\widetilde{\varphi }}_{A}^{k}$ as the $k$-th element of the null distribution representing the random variable ${\varPhi }_{A}$; (2) same for condition $B$, returning ${\widetilde{\varphi }}_{B}^{k}$ to form the null distribution of ${\varPhi }_{B}$; and (3) keeping the original cell-type labels but shuffling the $A$ and $B$ condition labels and returning the random score difference ${\delta }^{k}={\varphi }_{\widetilde{B}}^{k}-{\varphi }_{\widetilde{A}}^{k}$ to form the null distribution of the random variable $\Delta$. After iterating, the true values ${\varphi }_{A}$, ${\varphi }_{B}$ and $\delta ={\varphi }_{B}-{\varphi }_{A}$ are compared to the three null distributions to compute the two one-sided specificity *P* values ${p}_{A}=P({\varPhi }_{A} > {\varphi }_{A})$ and ${p}_{B}=P({\varPhi }_{B} > {\varphi }_{B})$ and the differential two-sided *P* value ${p}_{{DE}}=P(\left|\Delta \right| > \left|\delta \right|)$. Those *P* values are then adjusted for false discovery rate according to the Benjamini–Hochberg procedure^114^.

A CCI is considered ‘detected’ in condition $j\in \{A,B\}$ if (1) it is ‘expressed’; (2) it is ‘specific’, based on the specificity *P* values (${p}_{j}^{{adj}.}\le 0.05$); and (3) its score is among the top 80% of all the ‘specific’ CCI scores of both conditions, namely ${\varphi }_{j}\ge {q}_{{\varphi }_{A,B}}(20)$, where ${q}_{{\varphi }_{A,B}}(x)$ is the x-th percentile of the scores. A CCI is called ‘differentially expressed’ if ${p}_{{DE}}^{{adj}.}\le 0.05$ and $|\log ({\varphi }_{B}/{\varphi }_{A})|\ge \log (1.5)$.

As performing permutation tests can be computationally demanding, special care was taken to optimize the above computations. In particular, sDiffCom relies on the R package data.table (version 1.14.8)^115^ for fast manipulations of large data. Along the same line, we leveraged the R package future (version 1.32.0)^116^ allowing scDiffCom to perform permutation iterations in parallel. A toy model analysis (1,000 iterations on a dataset of 1,000 cells and five cell types) took a couple of minutes when run sequentially on a single-core computer. A more realistic example (10,000 permutations on 3,107 cells and 16 cell types) was measured to take around 9 min when run in parallel on 30 CPUs.

### CCI classification

We only kept CCIs that were ‘detected’ in at least one of the two conditions. They are then classified into four categories: (1) UP when ${p}_{{DE}}^{{adj}.}\le 0.05$ and ${logfc}\ge \log (1.5)$; (2) DOWN when ${p}_{{DE}}^{{adj}.}\le 0.05$ and ${logfc}\le -\log (1.5)$; (3) FLAT when $|{logfc}| < \log (1.5)$; and (4) NSC when ${p}_{{DE}}^{{adj}.} > 0.05$ and $|{logfc}|\ge \log (1.5)$.

The detection analysis was used to remove biologically irrelevant interactions but not to predict actual changes. Using the detection test for this purpose was indeed prone to return false-positive varying signals—that is, CCIs that seem to appear or disappear because they fluctuate around the detection threshold but that are, in reality, not differentially expressed. Using the aging datasets as benchmarking data, we considered all possible outcomes and noticed only a marginal number of seemingly contradictory cases between the two tests, such as disappearing CCIs with positive log fold changes (Supplementary Table 11). Those are, for instance, due to a reduction of the fraction of expressing cells, despite the increase of the signal, hence our decision to prioritize the classification of the CCIs based on the differential test that shuffles the $A$ and $B$ condition labels as explained in the previous subsection.

For benchmarking purposes, scDiffCom also performs standard differential expression analysis on individual genes in their respective cell type. Following the same notation as above, scDiffCom builds a distribution for the difference in expression of the ligand in emitter cells, $${E}_{L,B}-{E}_{L,A}$$, alongside the same permutation loop as for the CCI scores (similarly for the receptor $$R$$ in receiver cells). The *P* value for the ligand to be differentially expressed is computed as $${p}_{L,{DE}}=P(\left|{E}_{L,B}-{E}_{L,A}\right| > \left|{e}_{L,B}-{e}_{L,A}\right|)$$ and then adjusted according to Benjamini–Hochberg (similarly for the receptor $$R)$$). There are more direct methods than a permutation test (for example, a Wilcoxon test) to obtain such differential *P* values. However, our approach ensures that comparing these *P* values to the ‘CCI score *P* value’ does not suffer from technical biases as they are computed from the same permutations. In practice, we performed such a comparison using the 393,035 CCIs from scAgeCom. Using $${p}_{L,{DE}}^{{adj}.} < 0.05$$ and $$|{logf}{c}_{L}|\ge \log (1.5)$$ as thresholds for differential expression of the ligands (respectively, receptors), we saw that the classification of a considerable fraction of CCIs was determined without ambiguity by scDiffCom but not by standard differential expression (Extended Data Fig. 2).

### Fisher’s exact test to find over-represented signals

ORA is used to evaluate frequent patterns in categorical data—for example, to find if a particular feature of CCIs—for example, the annotation with the GO term ‘T cell differentiation’ is more frequent in upregulated CCIs compared to all other CCIs. This statistical association is measured by compiling the corresponding 2 × 2 contingency table (upregulated/not upregulated versus annotated/not annotated) and applying two-sided Fisher’s exact test. We performed this procedure for every CCI feature (all GO terms, KEGG pathways, LRIs, ligands, receptors and cell types) and classes (UP, DOWN and FLAT). It returns an OR and a *P* value adjusted for multiple testing according to the Benjamini–Hochberg procedure^114^. In some instances (for example, pattern ranking and plots), to sort the results based on a single value, we combined the OR and the *P* value to create an ORA score, by adapting the gene significance score (π value) used in differential gene expression analysis and gene set enrichment analysis^117^: ${ORA}\,{score}={\log }_{2}\,{OR}\cdot (-{lo}{g}_{10}\,{pval})$.

### Visualization tools in scDiffCom

We implemented two functions in scDiffCom to visualize the over-representation results. scDiffCom::PlotORA displays the top over-represented keywords of a given category and regulation. It is implemented on top of the R package ggplot2 (version 3.4.2)^118^. scDiffCom::BuildNetwork shows on a summary graph the over-represented cell types and cell-type pairs. It relies on the R package igraph (version 1.4.2)^119^ for internal computations and on the R package visNetwork (version 2.1.2)^120^ for the interactive rendering.

### Retrieving and preparing scRNA-seq datasets

We downloaded the latest version (as of 21 March 2021) of TMS from the Amazon S3 czb-tabula-muris-senis repository and the Calico dataset from the calicolabs website. They had been pre-processed and annotated with the Python toolkit Scanpy^121^ before our work, and we converted the resulting h5ad files to R Seurat objects.

As stated in the original TMS article^41^, FACS and Droplet refer to the technique used to capture the cells, namely (1) cell sorting in microtiter well plates followed by Smart-seq2 library preparation and full-length sequencing and (2) cell capture by microfluidic droplets as per the 10x Genomics protocol followed by 3′ end counting. The Calico data were exclusively obtained using the Droplet technique. Regarding mice age, TMS provided multiple timepoints that needed to be grouped into ‘young’ and ‘old’ categories. We removed 1-month-old cells and 30-month-old cells to avoid bias due to developmental or longevity-related processes. Therefore, we compared 3-month-old cells to 18/24-month-old cells from TMS and 7/8-month-old cells to 22/23-month-old cells from Calico. Finally, we filtered out tissues that were missing one age group (for example, TMS Droplet Fat contained only old cells).

The cells from each dataset were sequenced together. However, we decided to regroup TMS FACS Brain_Myeloid and Brain_Non-myeloid as the former contained only two cell types (macrophage and microglial cell), and merging the datasets allowed us to infer interactions with the other parts of the brain. We verified that this did not considerably alter the interactions detected in each dataset independently.

### Cell-type characterization

In each dataset, we standardized the names of the cell types based on Cell Ontology standards^122^—for example, ‘atrial myocyte’ was renamed as ‘regular atrial cardiac myocyte’. We also regrouped some specialized cell clusters—for example, CD4^+^ and CD8^+^ T cells—to increase sample size and avoid overlapping cell types. These overlaps were exceptionally kept in some tissues—for instance, the ‘undetermined myeloid leukocytes’ in the Lung dataset from Calico Droplet (male) overlap with some specialized cell types, such as ‘classical monocytes’, but were kept as distinct categories. Finally, we classified the cell types into 10 families to facilitate downstream analyses: ‘endothelial cells’, ‘epithelial cells’, ‘connective tissue cells’, ‘leukocytes’, ‘stem cells’, ‘neurons’, ‘glial cells’, ‘muscle cells’, ‘erythroid lineage cells’ and ‘hematopoietic precursor cells’ (Supplementary Table 12).

### Building and deploying scAgeComShiny

We used the R package golem (version 0.4.0)^123^ to build the Shiny app scAgeComShiny, which contains all scAgeCom results. Interactive scatter plots were built with plotly (version 4.10.1)^124^, which was also used to display the GO terms tree maps. Those were internally computed with the R package rrvgo (version 1.10.0)^125^, according to the original method from REVIGO^126^. To deploy the application, we first used golem to create a Docker image of the scAgeComShiny app and then serve it with the containerized version of ShinyProxy open-source middleware (version 3.0.1).

### Comparing scDiffCom to secretomics data

We leveraged the supplementary data of six recent studies to retrieve datasets of proteins secreted by six different cell types: mBMM^61^, mNeuron^62^, mMSC-AT^63^, rCM^64^, hUVEC^65^ and hPDE^66^. When necessary, we only kept the proteins secreted by control cells and discarded results related to specific conditions, such as cancer cells. Protein names were converted to MGI gene symbols (using orthology conversion if necessary) and intersected with the genes from our mouse LRI database. For each secretomics dataset, remaining gene symbols were used as input to an over-representation analysis assessing the cell-type specificity of the CCIs returned by scDiffCom. More precisely, each CCI was categorized into one of four groups depending on whether its ligand gene was part of the detected proteins and if its emitter cell type (or cell type family) was part of the cell type of interest. A two-sided Fisher’s exact test was applied to each such contingency table to compute corresponding *P* values and ORs. *P* values were further adjusted for multiple comparisons (Benjamini–Hochberg procedure). We focused our attention on the ligand and emitter parts of each CCI, as secretomics data do not directly provide information regarding the targets of secreted proteins.

### Statistics and reproducibility

No statistical method was used to pre-determine sample size. Sample size selection was informed and restricted by the availability of the public scRNA-seq data used in this study^41,42^. Randomization and blinding were not performed in this study, as all data collected were public, and the authors performing the analyses had already worked on most of the data before starting this study. Few groups of cells were excluded from the original scRNA-seq datasets to avoid biases, as explained in Methods. Both statistical tests implemented in scDiffCom (differential expression and over-representation) are non-parametric and do not assume the data to follow specific distributions. Other statistical tests are mentioned in the figures with additional details provided in Methods. Adjustments for multiple testing were always performed when relevant, as reported.

### Reporting summary

Further information on research design is available in the Nature Portfolio Reporting Summary linked to this article.

### Supplementary information

Supplementary information Supplementary Texts 1–4, Figs. 1–4 and References.

Reporting Summary

Supplementary Tables.

### Source data

Source Data Fig. 5 Statistical source data.

Source Data Fig. 6 Statistical source data.

Source Data Extended Data Fig. 1 Statistical source data.

Source Data Extended Data Fig. 2 Statistical source data.

## Data Availability

Source data are provided with this paper Datasets used in this study: • scRNA-seq Tabula Muris Senis: https://s3.console.aws.amazon.com/s3/buckets/czb-tabula-muris-senis • scRNA-seq Calico murine cell atlas: https://mca.research.calicolabs.com/ • LRIs from CellChat: available from their R package • LRIs from NicheNet: available from their R package • LRIs from SingleCellSignalR: available from their R package • LRIs from CellPhoneDB: https://www.cellphonedb.org/ • LRIs from CellTalkDB: https://github.com/ZJUFanLab/CellTalkDB • LRIs from connectomeDB2020: https://asrhou.github.io/NATMI/ • LRIs from ICELLNET: https://github.com/soumelis-lab/ICELLNET • General gene information: https://mygene.info/ and https://omnipathdb.org/ • GO terms: Ensembl (version 102), https://useast.ensembl.org/index.html • KEGG: accessed via the R package KEGGREST • gene2pubmed table: ftp://ftp.ncbi.nlm.nih.gov/gene/DATA/gene2pubmed.gz • GenAge (build 20): https://genomics.senescence.info/genes/index.html • LongevityMap (build 3): https://genomics.senescence.info/longevity/ • Microarray meta-analysis of Ageing Gene Expression database: https://genomics.senescence.info/gene_expression/ • CellAge (build 2): Supplementary Material of 10.1093/molbev/msab369 • Secretomics data for mBMM: Supplementary Material of 10.1126/science.1232578 • Secretomics data for mNeuron: Supplementary Material of 10.15252/embj.2020105693 • Secretomics data for mMSC-AT: Supplementary Material of 10.18632/aging.202423 • Secretomics data for rCM: Supplementary Material of 10.1161/circulationaha.119.044914 • Secretomics data for hUVEC: Supplementary Material of 10.1016/j.ajpath.2019.10.007 • Secretomics data for hPDE: Supplementary Material of 10.1002/pmic.202100320 Datasets created in this study: • scDiffCom LRIs: available from the R package scDiffCom (https://github.com/CyrilLagger/scDiffCom/tree/master/data) • Aging and sex scAgeCom results: 10.6084/m9.figshare.17074964 • Data to run the app scAgeComShiny and scagecom.org: https://figshare.com/articles/dataset/scAgeComShiny_data/17075375

## Code availability

All code used in this study is available from the following repositories:

• R package scDiffCom: https://github.com/CyrilLagger/scDiffCom

• R scripts for the aging/sex analyses: https://github.com/CyrilLagger/scAgeCom

• Golem package for the Shiny app: https://github.com/CyrilLagger/scAgeComShiny

• Docker image of the Shiny app: https://hub.docker.com/r/ursueugen/scagecom

Although scagecom.org is available online, we note that the content of the website can always be reproduced locally by installing our Shiny app from R as follows: devtools::install_github(‘CyrilLagger/scAgeComShiny’). The app can then be launched after having downloaded the necessary data in the app directory (https://figshare.com/articles/dataset/scAgeComShiny_data/17075375).

## References

[1] de Magalhães JP, Costa J (2009). A database of vertebrate longevity records and their relation to other life-history traits. J. Evol. Biol..

[2] López-Otín C, Blasco MA, Partridge L, Serrano M, Kroemer G (2013). The hallmarks of aging. Cell.

[3] Miller HA, Dean ES, Pletcher SD, Leiser SF (2020). Cell non-autonomous regulation of health and longevity. eLife.

[4] Capp, J.-P. & Thomas, F. Tissue-disruption-induced cellular stochasticity and epigenetic drift: common origins of aging and cancer? *Bioessays* **43**, e2000140 (2021). 10.1002/bies.202000140 33118188

[5] Franceschi C, Garagnani P, Parini P, Giuliani C, Santoro A (2018). Inflammaging: a new immune-metabolic viewpoint for age-related diseases. Nat. Rev. Endocrinol..

[6] Ovadya Y (2018). Impaired immune surveillance accelerates accumulation of senescent cells and aging. Nat. Commun..

[7] Fafián-Labora JA, O’Loghlen A (2020). Classical and nonclassical intercellular communication in senescence and ageing. Trends Cell Biol..

[8] Signer RAJ, Morrison SJ (2013). Mechanisms that regulate stem cell aging and life span. Cell Stem Cell.

[9] Oh J, Lee YD, Wagers AJ (2014). Stem cell aging: mechanisms, regulators and therapeutic opportunities. Nat. Med..

[10] Kurtz A, Oh S-J (2012). Age related changes of the extracellular matrix and stem cell maintenance. Prev. Med..

[11] Kehlet SN (2018). Age-related collagen turnover of the interstitial matrix and basement membrane: implications of age- and sex-dependent remodeling of the extracellular matrix. PLoS ONE.

[12] Russell SJ, Kahn CR (2007). Endocrine regulation of ageing. Nat. Rev. Mol. Cell Biol..

[13] Tatar M, Bartke A, Antebi A (2003). The endocrine regulation of aging by insulin-like signals. Science.

[14] Bartke A (2019). Growth hormone and aging: updated review. World J. Mens Health.

[15] Rothwell PM (2012). Short-term effects of daily aspirin on cancer incidence, mortality, and non-vascular death: analysis of the time course of risks and benefits in 51 randomised controlled trials. Lancet.

[16] Strong R (2008). Nordihydroguaiaretic acid and aspirin increase lifespan of genetically heterogeneous male mice. Aging Cell.

[17] Rothwell PM (2011). Effect of daily aspirin on long-term risk of death due to cancer: analysis of individual patient data from randomised trials. Lancet.

[18] Conboy IM (2005). Rejuvenation of aged progenitor cells by exposure to a young systemic environment. Nature.

[19] Conboy IM, Rando TA (2012). Heterochronic parabiosis for the study of the effects of aging on stem cells and their niches. Cell Cycle.

[20] Villeda SA (2014). Young blood reverses age-related impairments in cognitive function and synaptic plasticity in mice. Nat. Med..

[21] Raposo G, Stahl PD (2019). Extracellular vesicles: a new communication paradigm?. Nat. Rev. Mol. Cell Biol..

[22] Al Amir Dache Z (2020). Blood contains circulating cell-free respiratory competent mitochondria. FASEB J..

[23] Armingol E, Officer A, Harismendy O, Lewis NE (2021). Deciphering cell–cell interactions and communication from gene expression. Nat. Rev. Genet..

[24] Shao X, Lu X, Liao J, Chen H, Fan X (2020). New avenues for systematically inferring cell–cell communication: through single-cell transcriptomics data. Protein Cell.

[25] Ramilowski JA (2015). A draft network of ligand–receptor-mediated multicellular signalling in human. Nat. Commun..

[26] Jin S (2021). Inference and analysis of cell–cell communication using CellChat. Nat. Commun..

[27] Efremova M, Vento-Tormo M, Teichmann SA, Vento-Tormo R (2020). CellPhoneDB: inferring cell–cell communication from combined expression of multi-subunit ligand–receptor complexes. Nat. Protoc..

[28] Shao X (2021). CellTalkDB: a manually curated database of ligand–receptor interactions in humans and mice. Brief. Bioinformatics.

[29] Hou R, Denisenko E, Ong HT, Ramilowski JA, Forrest ARR (2020). Predicting cell-to-cell communication networks using NATMI. Nat. Commun..

[30] Noël F (2021). Dissection of intercellular communication using the transcriptome-based framework ICELLNET. Nat. Commun..

[31] Browaeys R, Saelens W, Saeys Y (2020). NicheNet: modeling intercellular communication by linking ligands to target genes. Nat. Methods.

[32] Cabello-Aguilar S (2020). SingleCellSignalR: inference of intercellular networks from single-cell transcriptomics. Nucleic Acids Res..

[33] Tsuyuzaki, K., Ishii, M. & Nikaido, I. Uncovering hypergraphs of cell–cell interaction from single cell RNA-sequencing data. Preprint at *bioRxiv*10.1101/566182 (2019).

[34] Solovey M, Scialdone A (2020). COMUNET: a tool to explore and visualize intercellular communication. Bioinformatics.

[35] He X, Memczak S, Qu J, Belmonte JCI, Liu G-H (2020). Single-cell omics in ageing: a young and growing field. Nat. Metab..

[36] Uyar B (2020). Single-cell analyses of aging, inflammation and senescence. Ageing Res. Rev..

[37] Ximerakis M (2019). Single-cell transcriptomic profiling of the aging mouse brain. Nat. Neurosci..

[38] Ma S (2020). Caloric restriction reprograms the single-cell transcriptional landscape of *Rattus norvegicus* aging. Cell.

[39] Ma S (2021). Single-cell transcriptomic atlas of primate cardiopulmonary aging. Cell Res..

[40] Zou Z (2021). A single-cell transcriptomic atlas of human skin aging. Dev. Cell.

[41] Tabula Muris Consortium. A single-cell transcriptomic atlas characterizes ageing tissues in the mouse. *Nature* **583**, 590–595 (2020). 10.1038/s41586-020-2496-1 PMC8240505 32669714

[42] Kimmel JC (2019). Murine single-cell RNA-seq reveals cell-identity- and tissue-specific trajectories of aging. Genome Res..

[43] Ben-Shlomo I, Yu Hsu S, Rauch R, Kowalski HW, Hsueh AJW (2003). Signaling receptome: a genomic and evolutionary perspective of plasma membrane receptors involved in signal transduction. Sci STKE.

[44] Prasad TSK, Kandasamy K, Pandey A (2009). Human Protein Reference Database and Human Proteinpedia as discovery tools for systems biology. Methods Mol. Biol..

[45] Harding SD (2018). The IUPHAR/BPS Guide to PHARMACOLOGY in 2018: updates and expansion to encompass the new guide to IMMUNOPHARMACOLOGY. Nucleic Acids Res..

[46] Fabregat A (2018). The Reactome Pathway Knowledgebase. Nucleic Acids Res..

[47] Kanehisa M, Furumichi M, Tanabe M, Sato Y, Morishima K (2017). KEGG: new perspectives on genomes, pathways, diseases and drugs. Nucleic Acids Res..

[48] Ashburner M (2000). Gene Ontology: tool for the unification of biology. Nat. Genet..

[49] Tacutu R (2018). Human Ageing Genomic Resources: new and updated databases. Nucleic Acids Res..

[50] Avelar RA (2020). A multidimensional systems biology analysis of cellular senescence in aging and disease. Genome Biol..

[51] Tejada-Martinez D (2022). Positive selection and enhancer evolution shaped lifespan and body mass in great apes. Mol. Biol. Evol..

[52] Budovsky A (2013). LongevityMap: a database of human genetic variants associated with longevity. Trends Genet..

[53] de Magalhães JP, Curado J, Church GM (2009). Meta-analysis of age-related gene expression profiles identifies common signatures of aging. Bioinformatics.

[54] Satija R, Farrell JA, Gennert D, Schier AF, Regev A (2015). Spatial reconstruction of single-cell gene expression data. Nat. Biotechnol..

[55] Butler A, Hoffman P, Smibert P, Papalexi E, Satija R (2018). Integrating single-cell transcriptomic data across different conditions, technologies, and species. Nat. Biotechnol..

[56] Stuart T (2019). Comprehensive integration of single-cell data. Cell.

[57] Büttner M, Ostner J, Müller CL, Theis FJ, Schubert B (2021). scCODA is a Bayesian model for compositional single-cell data analysis. Nat. Commun..

[58] Vento-Tormo R (2018). Single-cell reconstruction of the early maternal–fetal interface in humans. Nature.

[59] Good, P. *Permutation, Parametric and Bootstrap Tests of Hypotheses* (Springer, 2005).

[60] Wu T (2021). clusterProfiler 4.0: a universal enrichment tool for interpreting omics data. Innovation (Camb.).

[61] Meissner F, Scheltema RA, Mollenkopf H-J, Mann M (2013). Direct proteomic quantification of the secretome of activated immune cells. Science.

[62] Tüshaus J (2020). An optimized quantitative proteomics method establishes the cell type-resolved mouse brain secretome. EMBO J..

[63] Acar MB (2020). Obesity induced by high-fat diet is associated with critical changes in biological and molecular functions of mesenchymal stromal cells present in visceral adipose tissue. Aging (Albany, NY).

[64] Kuhn TC (2020). Secretome analysis of cardiomyocytes identifies PCSK6 (proprotein convertase subtilisin/kexin type 6) as a novel player in cardiac remodeling after myocardial infarction. Circulation.

[65] Zhao Y (2020). Quantitative proteomics of the endothelial secretome identifies RC0497 as diagnostic of acute rickettsial spotted fever infections. Am. J. Pathol..

[66] Li X (2022). Proteome and secretome analysis of pancreatic cancer cells. Proteomics.

[67] Ekpruke CD, Silveyra P (2022). Sex differences in airway remodeling and inflammation: clinical and biological factors. Front. Allergy.

[68] Ziegenhain C (2017). Comparative analysis of single-cell RNA sequencing methods. Mol. Cell.

[69] Zhang MJ, Pisco AO, Darmanis S, Zou J (2021). Mouse aging cell atlas analysis reveals global and cell type-specific aging signatures. eLife.

[70] Smith LK (2015). β2-microglobulin is a systemic pro-aging factor that impairs cognitive function and neurogenesis. Nat. Med..

[71] Castellano JM (2019). Blood-based therapies to combat aging. Gerontology.

[72] Basisty N (2020). A proteomic atlas of senescence-associated secretomes for aging biomarker development. PLoS Biol..

[73] Zhang Y (2019). Macrophage migration inhibitory factor rejuvenates aged human mesenchymal stem cells and improves myocardial repair. Aging (Albany, NY).

[74] Xia W, Zhang F, Xie C, Jiang M, Hou M (2015). Macrophage migration inhibitory factor confers resistance to senescence through CD74-dependent AMPK-FOXO3a signaling in mesenchymal stem cells. Stem Cell Res. Ther..

[75] Kokkola R (2005). RAGE is the major receptor for the proinflammatory activity of HMGB1 in rodent macrophages. Scand. J. Immunol..

[76] Abeyama K (2005). The N-terminal domain of thrombomodulin sequesters high-mobility group-B1 protein, a novel antiinflammatory mechanism. J. Clin. Invest..

[77] Doumas S, Kolokotronis A, Stefanopoulos P (2005). Anti-inflammatory and antimicrobial roles of secretory leukocyte protease inhibitor. Infect. Immun..

[78] Py B (2009). The phospholipid scramblases 1 and 4 are cellular receptors for the secretory leukocyte protease inhibitor and interact with CD4 at the plasma membrane. PLoS ONE.

[79] Jergović M, Contreras NA, Nikolich-Žugich J (2019). Impact of CMV upon immune aging: facts and fiction. Med. Microbiol. Immunol..

[80] Johnson AA, Stolzing A (2019). The role of lipid metabolism in aging, lifespan regulation, and age-related disease. Aging Cell.

[81] Riedel BC, Thompson PM, Brinton RD (2016). Age, APOE and sex: triad of risk of Alzheimer’s disease. J. Steroid Biochem. Mol. Biol..

[82] Chow VW, Mattson MP, Wong PC, Gleichmann M (2010). An overview of APP processing enzymes and products. Neuromolecul. Med..

[83] Yu J-T, Tan L, Hardy J (2014). Apolipoprotein E in Alzheimer’s disease: an update. Annu. Rev. Neurosci..

[84] Holtzman DM, Herz J, Bu G (2012). Apolipoprotein E and apolipoprotein E receptors: normal biology and roles in Alzheimer disease. Cold Spring Harb. Perspect. Med..

[85] Herz J, Beffert U (2000). Apolipoprotein E receptors: linking brain development and Alzheimer’s disease. Nat. Rev. Neurosci..

[86] Puig KL, Combs CK (2013). Expression and function of APP and its metabolites outside the central nervous system. Exp. Gerontol..

[87] Lee Y-H, Martin JM, Maple RL, Tharp WG, Pratley RE (2009). Plasma amyloid-β peptide levels correlate with adipocyte amyloid precursor protein gene expression in obese individuals. Neuroendocrinology.

[88] Lee Y-H (2008). Amyloid precursor protein expression is upregulated in adipocytes in obesity. Obesity (Silver Spring).

[89] Herzog V, Kirfel G, Siemes C, Schmitz A (2004). Biological roles of APP in the epidermis. Eur. J. Cell Biol..

[90] Puig KL, Swigost AJ, Zhou X, Sens MA, Combs CK (2012). Amyloid precursor protein expression modulates intestine immune phenotype. J. Neuroimmune Pharmacol..

[91] Galloway S, Jian L, Johnsen R, Chew S, Mamo JCL (2007). β-Amyloid or its precursor protein is found in epithelial cells of the small intestine and is stimulated by high-fat feeding. J. Nutr. Biochem..

[92] Askanas V, Engel WK (2006). Inclusion-body myositis: a myodegenerative conformational disorder associated with Aβ, protein misfolding, and proteasome inhibition. Neurology.

[93] Wang P (2005). Defective neuromuscular synapses in mice lacking amyloid precursor protein (APP) and APP-like protein 2. J. Neurosci..

[94] Borghesan M, O’Loghlen A (2017). Integrins in senescence and aging. Cell Cycle.

[95] de Magalhães JP (2023). Ageing as a software design flaw. Genome Biol..

[96] Moriya J, Minamino T (2017). Angiogenesis, cancer, and vascular aging. Front. Cardiovasc. Med..

[97] Hodges NA, Suarez-Martinez AD, Murfee WL (2018). Understanding angiogenesis during aging: opportunities for discoveries and new models. J. Appl. Physiol..

[98] Ambrose CT (2017). Pro-angiogenesis therapy and aging: a mini-review. Gerontology.

[99] Funasaka T, Haga A, Raz A, Nagase H (2001). Tumor autocrine motility factor is an angiogenic factor that stimulates endothelial cell motility. Biochem. Biophys. Res. Commun..

[100] Hayashi S-I, Rakugi H, Morishita R (2020). Insight into the role of angiopoietins in ageing-associated diseases. Cells.

[101] Tweedie S (2021). Genenames.org: the HGNC and VGNC resources in 2021. Nucleic Acids Res..

[102] Bult CJ (2019). Mouse Genome Database (MGD) 2019. Nucleic Acids Res..

[103] Yates AD (2020). Ensembl 2020. Nucleic Acids Res..

[104] Durinck S, Spellman PT, Birney E, Huber W (2009). Mapping identifiers for the integration of genomic datasets with the R/Bioconductor package biomaRt. Nat. Protoc..

[105] Xin J (2016). High-performance web services for querying gene and variant annotation. Genome Biol..

[106] Wu C, Macleod I, Su AI (2013). BioGPS and MyGene.info: organizing online, gene-centric information. Nucleic Acids Res..

[107] Türei D (2021). Integrated intra‐ and intercellular signaling knowledge for multicellular omics analysis. Mol. Syst. Biol..

[108] Loo MA (1998). Perturbation of Hsp90 interaction with nascent CFTR prevents its maturation and accelerates its degradation by the proteasome. EMBO J..

[109] Greene D, Richardson S, Turro E (2017). ontologyX: a suite of R packages for working with ontological data. Bioinformatics.

[110] Bioconductor. KEGGREST. 10.18129/B9.bioc.KEGGREST

[111] Winter DJ (2017). rentrez: an R package for the NCBI eUtils API. R J..

[112] Quinn TP, Erb I, Richardson MF, Crowley TM (2018). Understanding sequencing data as compositions: an outlook and review. Bioinformatics.

[113] Phillips, R., Kondev, J., Theriot, J. & Garcia, H. *Physical Biology of the Cell* (Garland Science, 2012).

[114] Benjamini Y, Hochberg Y (1995). Controlling the false discovery rate: a practical and powerful approach to multiple testing. J. R. Stat. Soc. Ser. B Methodol..

[115] Extension of ‘data.frame’. R package data.table version 1.14.8. https://cran.r-project.org/web/packages/data.table/index.html (2023).

[116] Bengtsson, H. Unified parallel and distributed processing in R for everyone. R package future version 1.32.0 (2023).

[117] Xiao Y (2014). A novel significance score for gene selection and ranking. Bioinformatics.

[118] Wickham, H. *ggplot2: Elegant Graphics for Data Analysis* (Springer, 2016).

[119] Csárdi, G. & Nepusz, T. The igraph software package for complex network research. *InterJournal* **1695**, 1–9 (2006).

[120] Almende, B. V., Thieurmel, B. & Robert, T. Network visualization using ‘vis.js’ library. R package visNetwork version 2.0.9 (2019).

[121] Wolf FA, Angerer P, Theis FJ (2018). SCANPY: large-scale single-cell gene expression data analysis. Genome Biol..

[122] Diehl AD (2016). The Cell Ontology 2016: enhanced content, modularization, and ontology interoperability. J. Biomed. Semantics.

[123] golem: a framework for robust Shiny applications. R package golem version 0.3.1. https://cran.r-project.org/web/packages/golem/index.html (2021).

[124] Plotly Technologies, Inc. *Collaborative Data Science* (Plotly Technologies, Inc., 2015).

[125] Sayols, S. rrvgo: a Bioconductor package for interpreting lists of Gene Ontology terms. *microPubl. Biol.* https://doi.org/doi:10.17912/micropub.biology.000811 (2023). 10.17912/micropub.biology.000811 PMC10155054 37151216

[126] Supek F, Bošnjak M, Škunca N, Šmuc T (2011). REVIGO summarizes and visualizes long lists of gene ontology terms. PLoS ONE.

