# Supplementary material for: scDiffCom: a tool for differential analysis of cell–cell interactions provides a mouse atlas of aging changes in intercellular communication
Source: Nat Aging. 2023 Nov 2;3(11):1446–61. doi: 10.1038/s43587-023-00514-x (PMC10645595; doi:10.1038/s43587-023-00514-x)
Supplement: Supplementary file 1 — Supplementary Texts 1–4, Figs. 1–4 and References. [file 43587_2023_514_MOESM1_ESM.pdf]

nature aging

Resource

https://doi.org/10.1038/s43587-023-00514-x

scDiffCom: a tool for differential analysis of cell–cell interactions provides a mouse atlas of aging changes in intercellular communication

In the format provided by the authors and unedited

Supplementary Information and Figures

Supplementary Text 1: sex differential analysis

Although studying the details of how intercellular communication depends on sex is beyond the scope of this work, we sought to apply scDiffCom to TMS datasets having both male and female samples to obtain CCIs differentially expressed with sex. We followed the same approach as for our aging analysis: we used the same parameters and considered young and old samples separately to avoid confounding factors.

In addition to being a resource for the community, these results allowed us to gain more information regarding potential sex dimorphisms in ICC age-related changes. We considered the CCIs differentially expressed between any of the four following conditions: young male, young female, old male, and old female (Supplementary Fig. 1). Keeping in mind that not all TMS tissues have cells in each category (Supplementary Table 8), we identified a total of 72,354 CCIs detected in all possible comparisons: young males vs young females (SY), old males vs young males (AM), old females vs young females (AF) and old males vs old females (SO). A simple consistency argument allowed us to ignore one of the four comparisons and we further only investigated the SY, AM, and AF cases. From the 72,354 detected CCIs, 24,068 CCIs are inferred as UP- or DOWN-regulated in at least one of the three comparisons – there are further referred to as changing CCIs (Supplementary Table 10). Most of the changing CCIs are only DOWN-regulated with age in males (~41%), while the fraction of DOWN-regulated CCIs with age only in females is significantly smaller (~4%). Most of the changing CCIs (~68%) are inferred as UP- or DOWN-regulated in exactly one of the 3 comparisons. From the other changing CCIs, those that are UP-regulated in SY and DOWN-regulated in AM are the predominant ones by a high margin (~13%) – these CCIs can be interpreted as highly-expressed in young males in comparison to both young females and old males (Supplementary Table 10). More specific patterns can be observed at the tissue-specific level. Describing all of them is beyond the scope of this study, but we report the case of the TMS FACS Lung in the main text of the manuscript in the section *Aging dysregulates multiple aspects of intercellular communication.*

Supplementary Text 2: GO enrichment analysis in scDiffCom

Absence of over-representation bias toward generic GO terms

As explained in the manuscript's main text, we annotated LRIs with the intersection of ligand and receptor ancestral GO terms. This method has for effect to attach generic terms (e.g. *biological_process*) to most LRIs. However, our overrepresentation analysis (ORA) results are not biased toward such GO terms. Indeed, being generic, these terms are attached to most CCIs and therefore have similar odds in both the group of interest and the background. For example, the answer to the question "Is the GO term *biological_process* more often observed in DOWN-regulated CCIs than in the background?" is always likely to be "NO" (with an odds ratio close

to 1 and a large, non-significant p-value). We confirmed this claim using scAgeCom results. For each dataset, we computed how the overrepresented GO terms are distributed according to their level, i.e. their depth in the GO graph (Supplementary Fig. 2a). The distribution of the medians over each dataset range from depth 5 to 7 with a peak at depth 6, confirming there is no bias towards low-depth terms. Along the same line, the distributions across datasets are similar to the distribution that is obtained by considering the set of all GO terms (Supplementary Fig. 2b).

Comparison of alternative GO annotation strategies

We sought to compare our GO annotation method to other "GO terms merging" strategies. Therefore, we implemented a feature in scDiffCom, enabling users to select the ligands and receptors GO terms merging mode. We define 8 such modes, 4 based on ancestors (ancestors intersection - the default, ancestors union, ancestors of ligands, ancestors of receptors) and 4 based on annotations ignoring ancestors (intersection, union, ligands, receptors). Further details are available online from our scDiffCom-GO vignette.

We tested these 8 methods by running scDiffCom on the liver toy-dataset sample available as part of our package. The distribution of GO levels (i.e. "depth" in the GO ontology graph) obtained by the overrepresentation analysis is similar across modes, meaning that none of them introduces any bias towards generic (low-level) GO terms (Supplementary Fig. 3a). However, the number of significant GO terms varies a lot across modes. This has to be expected from the fact that each mode will attach a different number of GO terms to each LRI (Supplementary Fig. 3b). For example, merging modes based on the union will attach more terms than merging modes based on the intersection. Similarly, considering the ancestors or not will lead to more, respectively less, GO terms. This pattern is apparent by looking at the actual numbers of GO terms attached to the LRIs (Supplementary Fig. 3c), and at how the distributions of median and maximal levels across the ligand-receptor interactions are influenced by the annotation method.

Ancestral intersection as the preferred GO terms annotation strategy

Overall, as the 8 modes do not introduce over-representation bias but change the total number of returned GO terms, choosing one of them is equivalent to defining which one is the most biologically relevant to the user. In our opinion, using the intersection of ancestors is the most suitable. First, considering ancestors is biologically relevant as relationships in the GO graph have the meaning of "is a" (e.g. *metabolic_process* "is a" *biological_process*). As such, the 4 modes that do not consider ancestors provide only a restricted annotation of each LRI. Second, choosing the intersection of ancestors (as opposed to the three other ancestor modes) ensures that we retain information relevant to both the ligand and the receptor. This is crucial because most gene products can have different functions depending on how they interact. In other words, relying on the intersection ensures that the annotation of two LRIs of the form geneA:geneB and geneA:geneC remains specific. On the contrary, using the union would associate the LRI geneA:geneB with functions that geneA might only have when it interacts with geneC.

Supplementary Text 3: Ligand-receptor Pairing and Coexpression

As most current ICC tools (e.g. CellChat and CellPhoneDB), scDiffCom relies on a prior database of curated LRIs. This ensures that any predicted CCI is mediated by a set of genes that have been confirmed to interact extracellularly. This is particularly important as our tool computes scores from scRNA-seq data, using intracellular mRNA expression as a proxy for actual protein secretion. As such, if we considered LRIs made of two random genes, some of them would still be detected as part of CCIs because their expression profile might look similar to actual ligands and receptors. This is why our detection method relies on both the curated LRIs and the permutation test ensuring cell-type specificity.

We still thought to compare the number of CCIs detected by scDiffCom when using either our curated LRI database or LRIs composed of random genes. For most TMS datasets, using the curated database leads to more detected CCIs (Supplementary Fig. 4a). We also checked how this impacts differential expression results (old vs young). Across TMS datasets, there are about 2-fold more UP or DOWN-regulated CCIs when using curated LRIs in contrast to using random pairing (Supplementary Fig. 4b). This might suggest that ligand and receptor genes are generally more differentially expressed with age than random genes. To test this hypothesis, we performed standard single-cell differential analysis on the TMS datasets (old vs young) and calculated the number and fraction of LRI genes among all differentially expressed genes (Supplementary Fig. 4c). Even though LRI genes constitute not more than ~9.1% of mouse protein-coding genes, the LRI fractions in the DGE genes across the TMS datasets are often >20% (Supplementary Fig. 4d). This suggests that LRI genes tend to be differentially expressed more than random genes.

We then explored coexpression among random gene pairs and genes LRIs. We leveraged the *high-confidence genes* subset from *CoCoCoNet* [1] as the data source for coexpression, thus obtaining mouse cross-tissue coexpression measurements. The authors measured gene pair coexpression based on normalized rank. The measure being derived from a rank makes the distribution of coexpression values to be uniform for random genes since the distribution of ranks is uniform (Supplementary Fig. 4e). This is not the case for the distribution of coexpression of LRI pairs (Supplementary Fig. 4f). Although the distribution shape for LRI pairs differs, with a median of 0.52 we did not find any evidence that their coexpression is higher or lower than that of random genes.

Supplementary Text 4: future considerations to Improve the Computation of CCI Scores

As high-throughput single-cell proteomics data are not easily accessible, scDiffCom currently relies on mRNA expression from scRNA-seq data to predict cell-cell interactions. Despite this limitation, we showed explicitly that our CCI score allows scDiffCom to detect interactions whose structure is globally consistent with secretomics data (Fig. 5 and Extended Data Fig. 1). Nevertheless, we could still be tempted to use prior knowledge in the formulation of the CCI

score to try to improve its accuracy. This typically includes parameters such as protein-RNA correlations or binding kinetics rate constants. Unfortunately, to the best of our knowledge, there currently exists no large-scale databases providing such reliable parameters for at least a significant fraction of all features/genes present in scRNA-seq data.

As such information might become available in the future, we discuss below two potential modifications to our CCI score. We remember that we are currently using the formula $\varphi = \sqrt{e_L e_R}$, based on the averaged expression e_L of the ligand gene in the emitter cells and the averaged expression e_R of the receptor gene in the receiver cells.

CCI score including protein-RNA correlation

A simple modification is to assume that protein level scales linearly with gene expression leading to the weighted CCI score $\varphi_w = \sqrt{w_L e_L w_R e_R}$ where the weights w represent protein-RNA correlations. Interestingly for the context of differential ICC analysis, we note that if the weights remain constant between two conditions A and B ($w_{L,A} = w_{L,B}$ and similarly for R), the log fold-change of the weighted CCI score is no different from the log-fold change of the usual score, $log\left(\frac{\varphi_{w,B}}{\varphi_{w,A}}\right) = log\left(\frac{\sqrt{e_{L,B}e_{R,B}}}{\sqrt{e_{L,A}e_{R,A}}}\right) = log\left(\frac{\varphi_B}{\varphi_A}\right)$, and gene expression would be sufficient to fully describe changes in ICC. However, realistically, we should expect protein-RNA correlation parameters to change between conditions as has been previously observed [2], notably during the aging process [3]. In other words, such a weighted score would only prove beneficial in our context if condition-specific protein-RNA correlations were available for a significant number of genes.

CCI score including binding kinetics

Our current CCI score does not include binding specificity. Inspired by binding kinetics considerations and Clark's equation [4], a modified score could be written $\varphi_{Clark} = \sqrt{e_R \frac{e_L}{e_L + K_D}}$ with K_D the dissociation constant specific to the ligand and receptor. Again, we are currently not aware of any database that provides a comprehensive number of reliable and condition-specific dissociation constants.

Supplementary Fig. 1

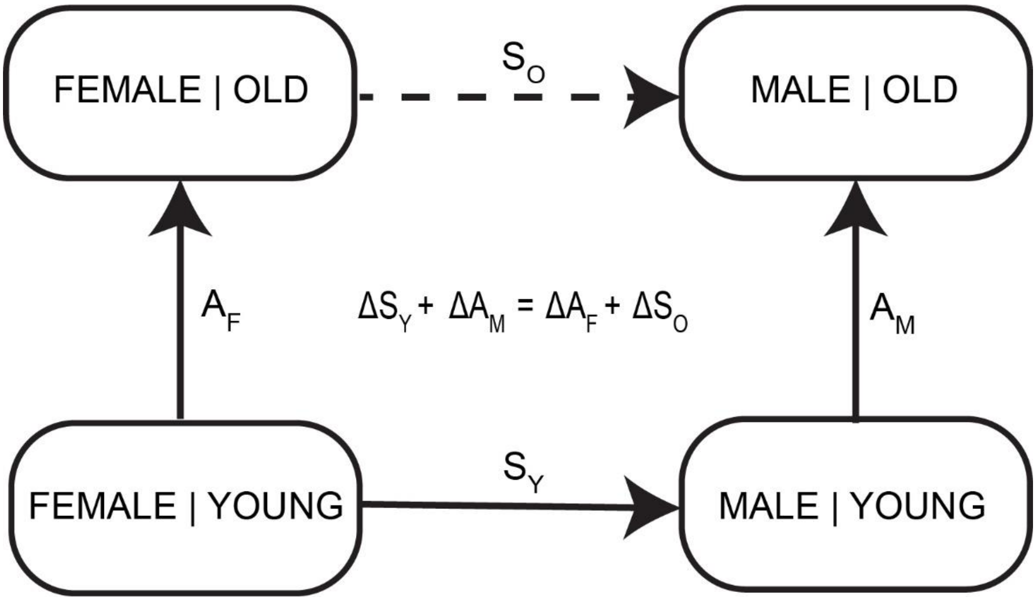

Relationships between sample types for the integration of the sex and aging analyses. There are four different states (young female, young male, old female, and old male) and we considered four comparisons: young male vs young female (SY - sex difference in youngs), young female vs old female (AF - age difference in females), old male vs young male (AM - age difference in male), and old male vs old female (SO - sex difference in olds). Denoting by Δi the log fold-change of the CCI score for the comparison i, we note that there exists a simple consistency relation between the four differences. Using scAgeCom results, we verified that this relation holds up to numerical errors of the order of 1e-15. Therefore, we focused on only three comparisons for our analyses described in Supplementary Text 1 (namely SY, AF, and AM).

Supplementary Fig. 2

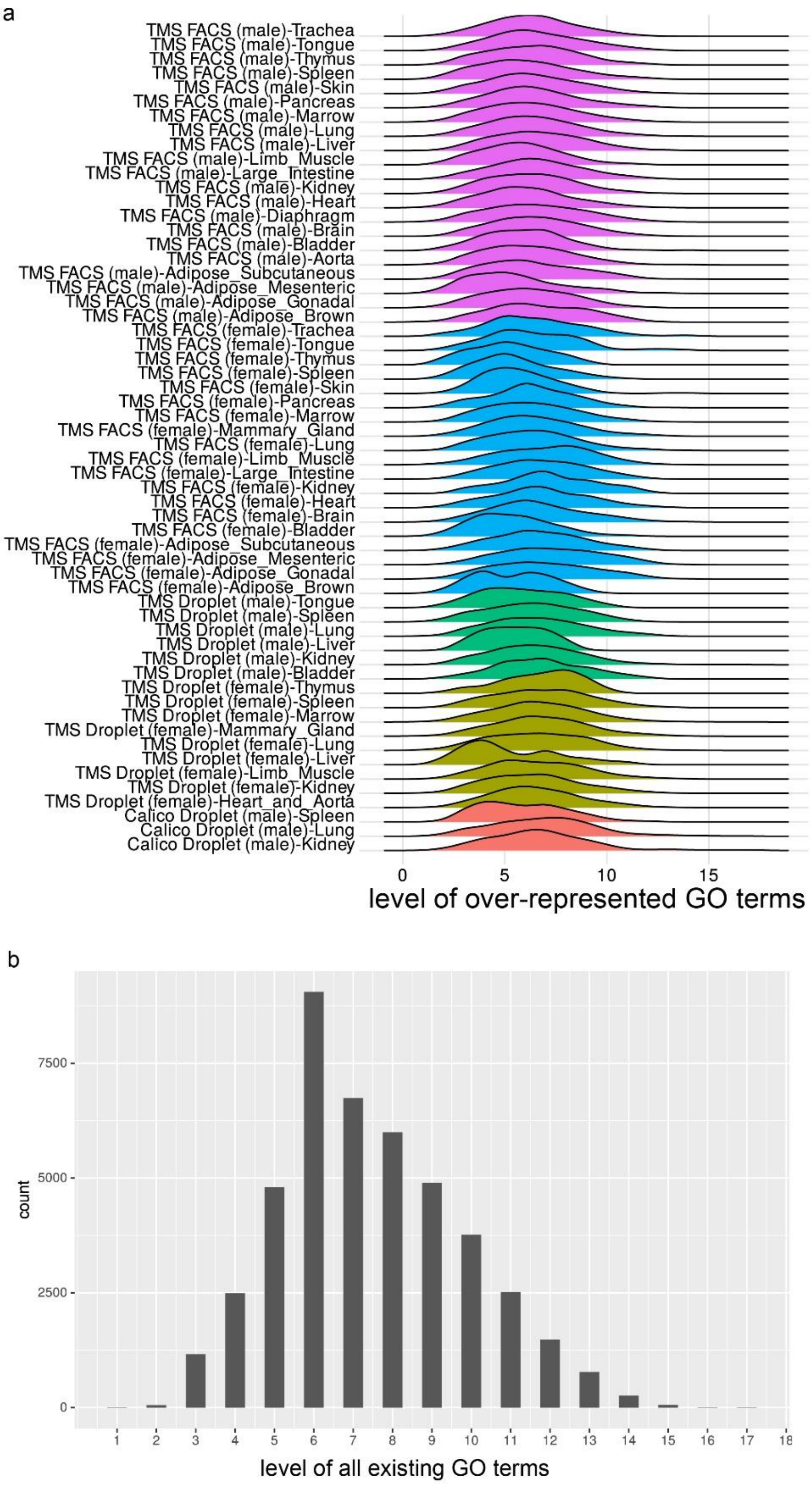

Over-represented GO terms are not biased toward low-depth terms. a, The distribution of levels (i.e. depth in the GO graph) of up and down over-represented GO terms is similar across all 58 datasets, with a median at level 6. **b,** The depth distribution of all existing GO terms (>44,000 terms) shows that most GO terms have a level between 5 and 10 with a peak at level 6. Overall, this confirms that the over-representation analysis (ORA) implemented in scDiffCom does not introduce biases in the level of over-represented GO terms.

Supplementary Fig. 3

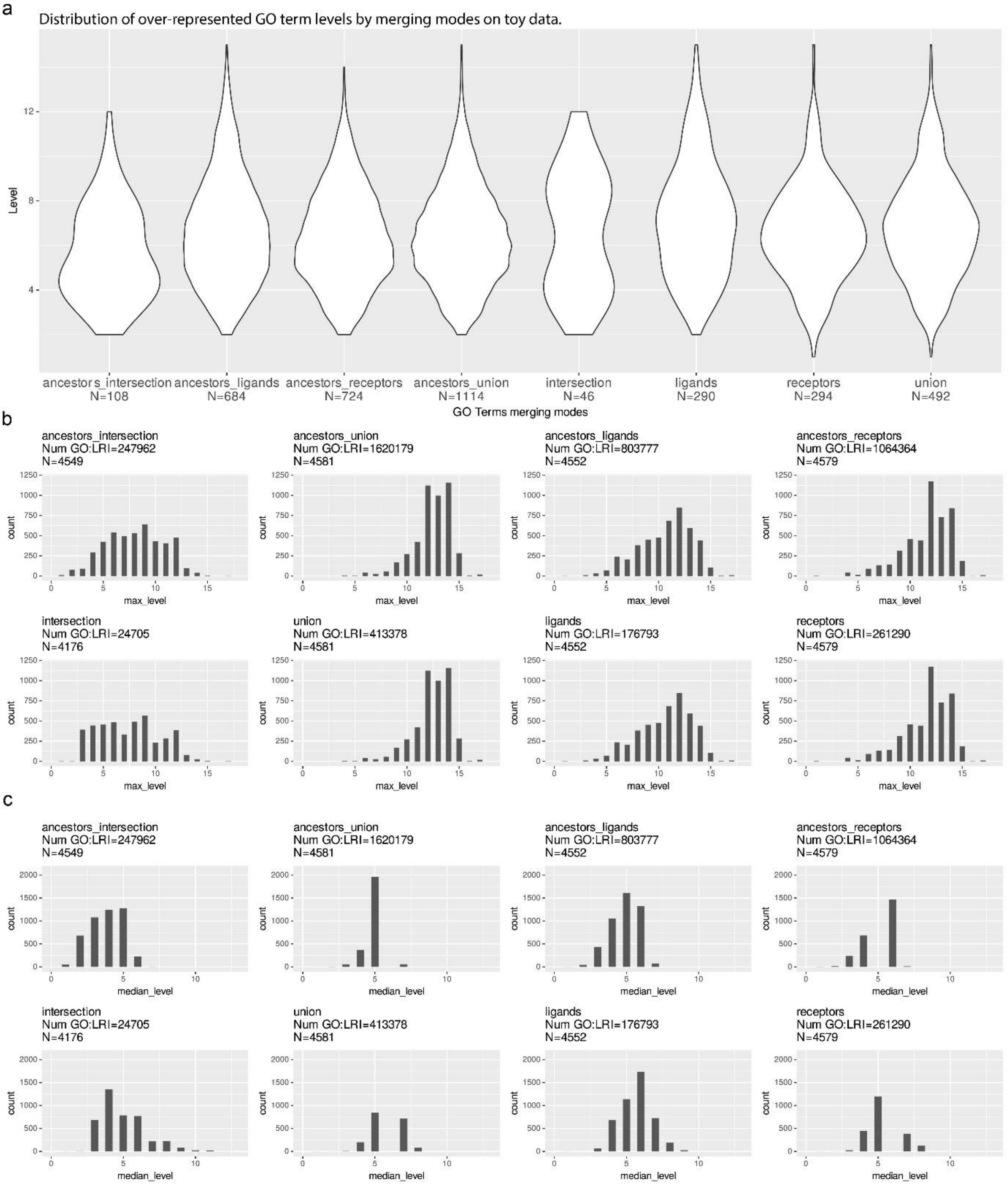

Comparison of 8 GO merging strategies to annotate LRIs. Each LRI from the scDiffCom database is assigned a set of GO terms based on various strategies (described in the text) that merge the GO information of the ligand and the receptor. **a,** When performing scDiffCom differential analysis on the liver toy scRNA-seq dataset provided as part of the package, and performing over-representation analysis, we noticed that the distribution of over-represented GO terms levels is similar across all 8 merging strategies. *N* represents the number of GO terms over-represented in each case. **b,c,** Distributions of maximum (b) and median (c) GO level for

each LRI across merging strategies. *Num GO:LRI* indicates the total number of annotations for a given strategy, namely the sum over LRIs of the number of GO terms annotated to each LRI. *N* indicates the number of LRIs with at least one annotation. The distribution of maxima is highly similar across the ancestors vs non-ancestors merging modes, as expected since maximal depth is invariant to considering ancestors or not.

Supplementary Fig. 4

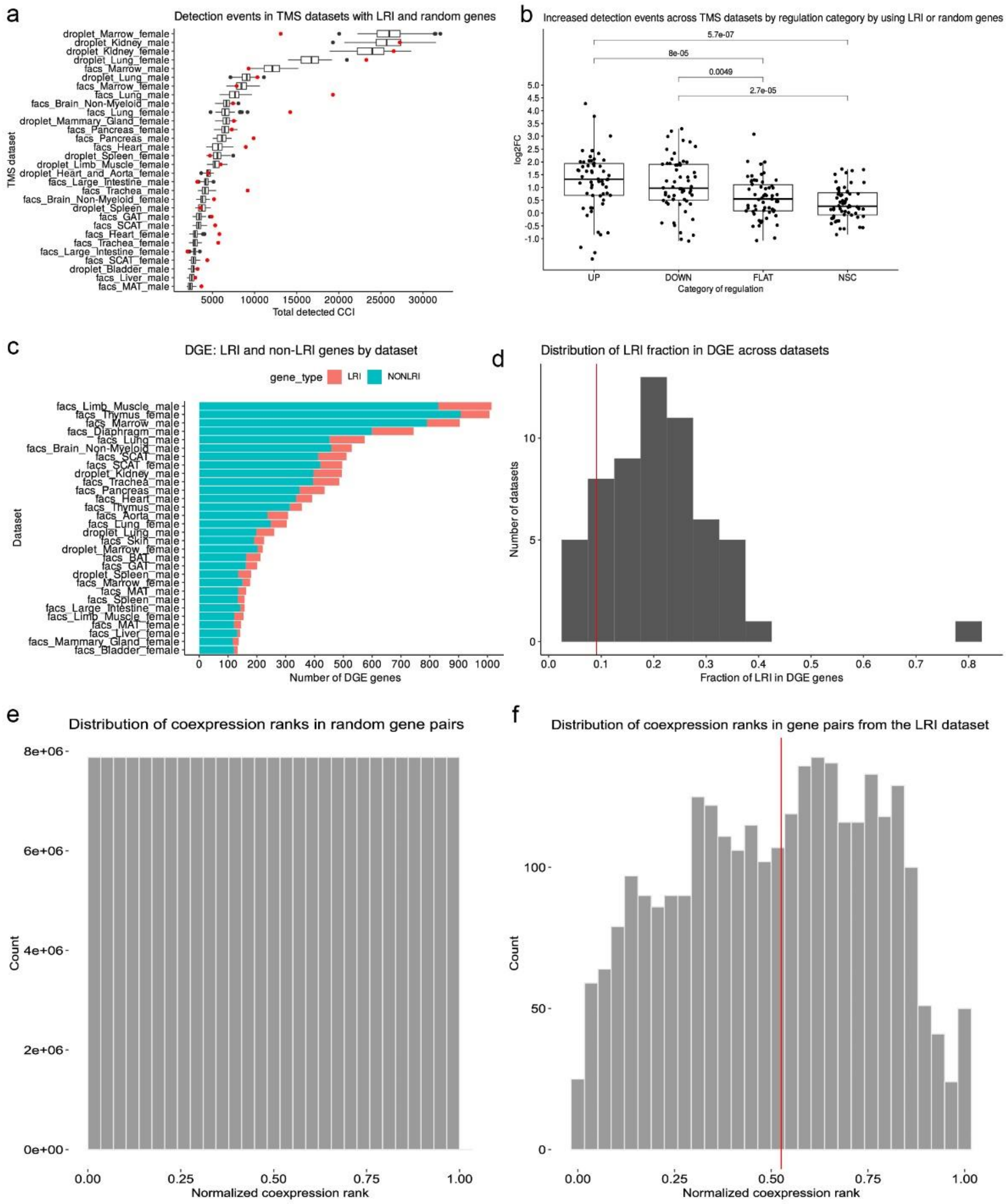

Analysis of random gene pairing and ligand-receptor coexpression. a, Number of CCIs detected by scDiffCom in several TMS datasets using as LRIs either random gene pairs (boxplot and black dots, N = 50 simulations) or curated LRIs from scDiffCom database (red dots). The box of the boxplot shows the interquartile range (IQR) and spans from the first quartile (Q1) to the third quartile (Q3), the middle line representing the median. The upper whisker extends to a maximum of 1.5 times the IQR above Q3, while the lower whisker extends to a minimum of 1.5 times the IQR below Q1. **b,** Fold change of the numbers of detected CCIs between using curated or random LRIs per regulation category (N = 58 datasets). There are ~2-fold more up and down-regulated CCIs when using curated LRIs as opposed to random LRIs. The box of the boxplot shows the interquartile range (IQR) and spans from the first quartile (Q1) to the third quartile (Q3), the middle line representing the median. The upper whisker extends to a maximum of 1.5 times the IQR above Q3, while the lower whisker extends to a

minimum of 1.5 times the IQR below Q1. Jittered data points are overlaid on the plot. P-values between categories of regulation were computed using a Wilcoxon signed-rank test. **c,** Number of genes differentially expressed with age in several TMS datasets and how many of them are either ligands or receptors. **d,** Distribution of the fraction of genes that are ligands or receptors among differentially expressed genes across TMS datasets. The red line indicates the fraction of genes that are ligands or receptors in the mouse genome. Most datasets have at least 20% of their DEGs that are ligands or receptors. **e,f,** Distribution of normalized coexpressions ranks for all genes (e) and curated LRI gene pairs (f). The vertical red line indicates the median of the distribution.

Supplementary References

1. Lee, J., Shah, M., Ballouz, S., Crow, M. & Gillis, J. CoCoCoNet: conserved and comparative co-expression across a diverse set of species. *Nucleic Acids Res.* **48**, W566–W571 (2020).
2. Wegler, C. *et al.* Global variability analysis of mRNA and protein concentrations across and within human tissues. *NAR Genom. Bioinform.* **2**, lqz010 (2020).
3. Wei, Y.-N. *et al.* Transcript and protein expression decoupling reveals RNA binding proteins and miRNAs as potential modulators of human aging. *Genome Biol.* **16**, 41 (2015).
4. Buchwald, P. A receptor model with binding affinity, activation efficacy, and signal amplification parameters for complex fractional response versus occupancy data. *Front. Pharmacol.* s**10**, 605 (2019).
