# Supplementary material for: scDiffCom: a tool for differential analysis of cell–cell interactions provides a mouse atlas of aging changes in intercellular communication
Source: Nat Aging. 2023 Nov 2;3(11):1446–61. doi: 10.1038/s43587-023-00514-x (PMC10645595; doi:10.1038/s43587-023-00514-x)
Supplement: Supplementary file 2 — Reporting Summary [file 43587_2023_514_MOESM2_ESM.pdf]

nature portfolio

Corresponding author(s): João Pedro de Magalhães

Last updated by author(s): Sep 6, 2023

Reporting Summary

Nature Portfolio wishes to improve the reproducibility of the work that we publish. This form provides structure for consistency and transparency in reporting. For further information on Nature Portfolio policies, see our Editorial Policies and the Editorial Policy Checklist.

Statistics

For all statistical analyses, confirm that the following items are present in the figure legend, table legend, main text, or Methods section.

n/a	Confirmed	
☐	☒	The exact sample size (*n*) for each experimental group/condition, given as a discrete number and unit of measurement
☒	☐	A statement on whether measurements were taken from distinct samples or whether the same sample was measured repeatedly
☐	☒	The statistical test(s) used AND whether they are one- or two-sided<br>*Only common tests should be described solely by name; describe more complex techniques in the Methods section.*
☐	☒	A description of all covariates tested
☐	☒	A description of any assumptions or corrections, such as tests of normality and adjustment for multiple comparisons
☐	☒	A full description of the statistical parameters including central tendency (e.g. means) or other basic estimates (e.g. regression coefficient) AND variation (e.g. standard deviation) or associated estimates of uncertainty (e.g. confidence intervals)
☐	☒	For null hypothesis testing, the test statistic (e.g. *F*, *t*, *r*) with confidence intervals, effect sizes, degrees of freedom and *P* value noted<br>*Give P values as exact values whenever suitable.*
☒	☐	For Bayesian analysis, information on the choice of priors and Markov chain Monte Carlo settings
☒	☐	For hierarchical and complex designs, identification of the appropriate level for tests and full reporting of outcomes
☒	☐	Estimates of effect sizes (e.g. Cohen's *d*, Pearson's *r*), indicating how they were calculated

Our web collection on statistics for biologists contains articles on many of the points above.

Software and code

Policy information about availability of computer code

Data collection	Some data were collected with the following software and packages: R(v4.1.0), rentrez (v1.2.3, R package), CellChat (v1.1.3, R package), NicheNet (v1.1.1, R package), SingleCellSignalR (v.1.10.0, R package).<br><br>Multiple datasets were also collected online from their respective websites (see Data section below).<br><br>The way each data type was collected is fully described in detail in our Methods section and/or in our github repositories: https://github.com/CyrilLagger/scDiffCom and https://github.com/CyrilLagger/scAgeCom.
Data analysis	Data analyses were performed with the following sofware/code: R(v4.1.0), scDiffCom (v0.2.4, R package), scAgeComShiny (v0.2, R package), data.table (v1.14.2, R package), biomaRt (v2.50.2, R package), rentrez (v1.2.3, R package), Seurat (v4.1.0, R package), org.Mm.eg.db (v3.14.0, R package), future (v1.25.0, R package), rrvgo (v1.6.0, R package), GOSemSim (v2.20.0, R package), ggplot2 (v3.3.6, R package), ComplexUpset (v1.3.3, R package), plotly (v4.10.0, R package), gt (v0.9.0, R package), ontoProc (v1.16.0, R package), KEGGREST (v1.34.0, R package), igraph (v1.2.11, R package), visNetwork (v2.1.0, R package).<br><br>scDiffCom and scAgeComShiny are two R packages we built for this study. They are available at https://github.com/CyrilLagger/scDiffCom, and https://github.com/CyrilLagger/scAgeComShiny, which also provide the full lists of their dependencies.<br><br>All scripts are available on github at: https://github.com/CyrilLagger/scAgeCom and https://github.com/CyrilLagger/scAgeComShiny.

For manuscripts utilizing custom algorithms or software that are central to the research but not yet described in published literature, software must be made available to editors and reviewers. We strongly encourage code deposition in a community repository (e.g. GitHub). See the Nature Portfolio guidelines for submitting code & software for further information.

Data

Policy information about availability of data

All manuscripts must include a data availability statement. This statement should provide the following information, where applicable:

- Accession codes, unique identifiers, or web links for publicly available datasets
- A description of any restrictions on data availability
- For clinical datasets or third party data, please ensure that the statement adheres to our policy

Datasets used in this study:
- scRNA-seq Tabula Muris Senis: https://s3.console.aws.amazon.com/s3/buckets/czb-tabula-muris-senis
- scRNA-seq Calico murine cell atlas: https://mca.research.calicolabs.com/
- LRIs from CellChat: available from the R package (see data collection)
- LRIs from NicheNet: available from the R package (see data collection)
- LRIs from SingleCellSignalR: available from the R package (see data collection)
- LRIs from CellPhoneDB: https://www.cellphonedb.org/
- LRIs from CellTalkDB: https://github.com/ZJUFanLab/CellTalkDB
- LRIs from connectomeDB2020: https://asrhou.github.io/NATMI/
- LRIs from ICELLNET: https://github.com/soumelis-lab/ICELLNET
- general gene information: https://mygene.info/ and https://omnipathdb.org/
- GO terms: Ensembl (v102) https://useast.ensembl.org/index.html
- KEGG: accessed via the R package KEGGREST (v1.34.0)
- gene2pubmed table: ftp://ftp.ncbi.nlm.nih.gov/gene/DATA/gene2pubmed.gz
- GenAge (build 20): https://genomics.senescence.info/genes/index.html
- LongevityMap (build 3): https://genomics.senescence.info/longevity/
- Microarray meta-analysis of Ageing Gene Expression database: https://genomics.senescence.info/gene_expression/
- CellAge (build 2): Supplementary Material of https://doi.org/10.1093/molbev/msab369
- secretomics data for mBMM: Supplementary Material of https://doi.org/10.1126/science.1232578
- secretomics data for mNeuron: Supplementary Material of https://doi.org/10.15252/embj.2020105693
- secretomics data for mMSC-AT: Supplementary Material of https://doi.org/10.18632/aging.202423
- secretomics data for rCM: Supplementary Material of https://doi.org/10.1161/circulationaha.119.044914
- secretomics data for hUVEC: Supplementary Material of https://doi.org/10.1016/j.ajpath.2019.10.007
- secretomics data for hPDE: Supplementary Material of https://doi.org/10.1002/pmic.202100320

Datasets created in this study:
- scDiffCom LRIs: available from the R package scDiffCom (https://github.com/CyrilLagger/scDiffCom/tree/master/data)
- aging and sex scAgeCom results: http://doi.org/10.6084/m9.figshare.17074964
- data to run the app scAgeComShiny and scagecom.org: https://figshare.com/articles/dataset/scAgeComShiny_data/17075375

Human research participants

Policy information about studies involving human research participants and Sex and Gender in Research.

Reporting on sex and gender	Our analysis studied murine age-related changes in intercellular communication from public scRNA-seq datasets that contain both male and female mouse samples. Therefore, we performed first an aging-differential analysis on male and female samples separately and second a sex-differential analysis on young and old samples separately. This allowed us two create both an age-related database and a sex-related database of intercellular communication changes that are publicly available (see data availability). As aging is the main focus of this study, we mainly focused on interpreting our age-related results but we also used our sex-related results to reveal sex-specific age-related changes in extracellular signalling.
Population characteristics	N/A
Recruitment	N/A
Ethics oversight	N/A

Note that full information on the approval of the study protocol must also be provided in the manuscript.

Field-specific reporting

Please select the one below that is the best fit for your research. If you are not sure, read the appropriate sections before making your selection.

☒ Life sciences ☐ Behavioural & social sciences ☐ Ecological, evolutionary & environmental sciences

For a reference copy of the document with all sections, see nature.com/documents/nr-reporting-summary-flat.pdf

Life sciences study design

All studies must disclose on these points even when the disclosure is negative.

Sample size	No statistical method was used to predetermine sample size. Sample size selection was informed and restricted by the availability of the public scRNA-seq data used in this study (ref 41,42).
Data exclusions	Few groups of cells were excluded from the original scRNA-seq datasets to avoid biases as explained in the Methods.
Replication	Experimental replication was not performed in this study as all data collected were public and no new experimental data have been generated. However, some of the results in our atlas have been obtained from several independent public datasets (TMS FACS, TMS Droplet and the Calico murine aging cell atlas) allowing to compare them.
Randomization	We controlled for sex in our aging analysis by treating male and female datasets separately. Likewise, we control for age in the sex analysis by treating young and old datasets separately.
Blinding	Blinding was not performed in this study as all data collected were public and the authors performing the analyses had already worked on most of the data before starting this study.

Reporting for specific materials, systems and methods

We require information from authors about some types of materials, experimental systems and methods used in many studies. Here, indicate whether each material, system or method listed is relevant to your study. If you are not sure if a list item applies to your research, read the appropriate section before selecting a response.

Materials & experimental systems

n/a	Involved in the study	
☒	☐	Antibodies
☒	☐	Eukaryotic cell lines
☒	☐	Palaeontology and archaeology
☒	☐	Animals and other organisms
☒	☐	Clinical data
☒	☐	Dual use research of concern

Methods

n/a	Involved in the study	
☒	☐	ChIP-seq
☒	☐	Flow cytometry
☒	☐	MRI-based neuroimaging
